# iTRAQ-Based Global Phosphoproteomics Reveals Novel Molecular Differences Between *Toxoplasma gondii* Strains of Different Genotypes

**DOI:** 10.3389/fcimb.2019.00307

**Published:** 2019-08-23

**Authors:** Ze-Xiang Wang, Chun-Xue Zhou, Guillermo Calderón-Mantilla, Evangelia Petsalaki, Jun-Jun He, Hai-Yang Song, Hany M. Elsheikha, Xing-Quan Zhu

**Affiliations:** ^1^State Key Laboratory of Veterinary Etiological Biology, Key Laboratory of Veterinary Parasitology of Gansu Province, Lanzhou Veterinary Research Institute, Chinese Academy of Agricultural Sciences, Lanzhou, China; ^2^College of Veterinary Medicine, Gansu Agricultural University, Lanzhou, China; ^3^Department of Parasitology, Shandong University School of Basic Medicine, Jinan, China; ^4^European Molecular Biology Laboratory, European Bioinformatics Institute, Wellcome Genome Campus, Hinxton, United Kingdom; ^5^College of Veterinary Medicine, Yunnan Agricultural University, Kunming, China; ^6^Faculty of Medicine and Health Sciences, School of Veterinary Medicine and Science, University of Nottingham, Sutton Bonington Campus, Loughborough, United Kingdom

**Keywords:** *Toxoplasma gondii*, genotype, tachyzoite, iTRAQ, phosphoproteomics

## Abstract

To gain insights into differences in the virulence among *T. gondii* strains at the post-translational level, we conducted a quantitative analysis of the phosphoproteome profile of *T. gondii* strains belonging to three different genotypes. Phosphopeptides from three strains, type I (RH strain), type II (PRU strain) and ToxoDB#9 (PYS strain), were enriched by titanium dioxide (TiO2) affinity chromatography and quantified using iTRAQ technology. A total of 1,441 phosphopeptides, 1,250 phosphorylation sites and 759 phosphoproteins were detected. In addition, 392, 298, and 436 differentially expressed phosphoproteins (DEPs) were identified in RH strain when comparing RH/PRU strains, in PRU strain when comparing PRU/PYS strains, and in PYS strain when comparing PYS/RH strains, respectively. Functional characterization of the DEPs using GO, KEGG, and STRING analyses revealed marked differences between the three strains. *In silico* kinase substrate motif analysis of the DEPs revealed three (RxxS, SxxE, and SxxxE), three (RxxS, SxxE, and SP), and five (SxxE, SP, SxE, LxRxxS, and RxxS) motifs in RH strain when comparing RH/PRU strains, in PRU strain when comparing PRU/PYS, and in PYS strain when comparing PYS/RH strains, respectively. This suggests that multiple overrepresented protein kinases including PKA, PKG, CKII, IKK, and MAPK could be involved in such a difference between *T. gondii* strains. Kinase associated network analysis showed that ROP5, ROP16, and cell-cycle-associated protein kinase CDK were the most connected kinase peptides. Our data reveal significant changes in the abundance of phosphoproteins between *T. gondii* genotypes, which explain some of the mechanisms that contribute to the virulence heterogeneity of this parasite.

## Introduction

*Toxoplasma gondii* is a strictly intracellular protozoan parasite, which can infect almost any warm-blooded animal and over two billion people, worldwide. *T. gondii* infection is generally mild and asymptomatic in healthy immunocompetent individuals, but can be life-threatening in patients with compromised immune system, such as HIV-infected persons and organ transplant recipients, and in fetuses infected *in utero* (Elsheikha, 2008). The three clonal lineages of *T. gondii* commonly found in Eurasia and North America (Type I, II, and II) vary greatly in terms of virulence and phenotypic plasticity (Howe and Sibley, 1995; Sibley and Ajioka, 2008; Sibley et al., 2009a; Khan et al., 2011; Pittman et al., 2014; Shwab et al., 2014). The type I strains are highly virulent for mice, but with limited ability to transform to bradyzoites (Sibley and Boothroyd, 1992; Saeij et al., 2005). Type III strains can transform to bradyzoites and induce chronic infection, but they rarely cause clinical illness in humans. Type II strains are intermediate between type I and type III (Cheng et al., 2015; Ivanova et al., 2016). The Chinese 1 (ToxoDB#9) is the predominant lineage in mainland China and accounts for 78% of total strains isolated from animals and humans (Jiang et al., 2013; Wang et al., 2013, 2015; Li et al., 2014).

Microarray and next generation DNA sequencing (NGS) technologies have been used to investigate genetic basis for phenotypic differences between different *T. gondii type* I strains (Yang et al., 2013). Also, two-dimensional difference gel electrophoresis (2D-DIGE) coupled with MALDI-TOF-MS was used to identify differentially expressed proteins among tachyzoites of different *T. gondii* genotypes (Zhou et al., 2014). The differential expression of proteins involved in the parasite virulence can even vary between virulent strains, such as Korean Isolate-1 (KI-1) and RH, of the same genotype (Choi et al., 2010). The role of protein phosphorylation in the mediation of virulence of *T. gondii* has been also reported (Joyce et al., 2011; Lorenzi et al., 2016). Protein phosphorylation is one of the most important post-translational protein modifications (PTMs) and functions in signal transduction pathways by modulating protein activity and protein-protein interactions (Pawson and Scott, 2005; Mithoe and Menke, 2011). Phosphorylation is regulated by protein kinases and protein phosphatases through their dynamically balanced actions. *T. gondii* genome consists of 8,311 ORFs, including 159 predicted eukaryotic-like protein kinases and harbors 108 active eukaryotic-like protein kinases (Peixoto et al., 2010; Lorenzi et al., 2016). Difference in virulence between *T. gondii* strains has been attributed to polymorphic variations in rhoptry (ROP) protein kinases and pseudokinases secreted by *T. gondii* strains to mediate distinct pathogenic processes in the host cell, such as virulence and modulation of host cell signaling (Saeij et al., 2006; Taylor et al., 2006; Khan et al., 2009; Sibley et al., 2009b; Behnke et al., 2011; Reese et al., 2011; Lim et al., 2012). A previous phosphoproteomic study reported that phosphorylation can regulate proteins that play critical role in the invasion and remodeling of the host cell and that *T. gondii* calcium-dependent protein kinase 1 (CDPK1) contains at least three major phosphosites (serine 25, 61, and 349) (Treeck et al., 2011). Analysis of kinase-deficient mutants *T. gondii* strains showed that protein phosphorylation plays an important role in the phosphorylation and activation of the host transcription factors STAT3 and STAT6 (Saeij et al., 2007; Ong et al., 2010), TgCDPK3-mediated parasite egress (Treeck et al., 2014), and efficient production of brain cysts (Sugi et al., 2017). Additionally, the rhoptry proteins ROP18 and ROP5 were found to mediate *T. gondii* evasion of the murine interferon-gamma response (Niedelman et al., 2012). The virulence of *T. gondii* Type I strains in mice is attributed to inactivation of host IRG resistance proteins by targeted phosphorylation of threonine residues in the switch I loop by ROP18 kinase (Steinfeldt et al., 2015). Therefore, studying the differences in the abundance of phosphoproteins between major genotypes of *T. gondii* can complement the picture derived from previous transcriptomic and proteomic profiling studies and provide new insight into the molecular mechanisms controlling parasite virulence.

Recent advances in quantitative mass spectrometry-based proteomics approaches, such as iTRAQ (Isobaric tag for relative and absolute quantitation) combined with phosphopeptide enrichment using titanium dioxide (TiO_2_) affinity chromatography have enabled the sensitive and accurate identification and characterization of the proteome and phosphoproteome of multiple samples simultaneously and in an unbiased manner (Becker and Bern, 2011; Borchert et al., 2012; Glibert et al., 2015; Solari et al., 2016; Amorim et al., 2017). In the present study, a quantitative phosphoproteomic approach using iTRAQ combined with TiO_2_ affinity chromatography was employed to characterize the phosphoproteomic differences between *T. gondii* tachyzoites of the three major genotypes. Our findings contribute a new resource for understanding strain-specific variations in molecular signaling pathways, which may underpin some of the mechanisms that contribute to the pathogenicity of *T. gondii*.

## Materials and Methods

### Parasite Preparation

Tachyzoites of Type I (RH) and Chinese 1 (PYS) *T. gondii* were recovered from frozen samples stored in liquid nitrogen as described previously (Zhou et al., 2014). Tachyzoites of each strain were inoculated into female, 6–8 weeks old, specific-pathogen-free (SPF) BALB/c mice in order to allow tachyzoites to revive. Mice were provided by Laboratory Animal Center of Lanzhou Veterinary Research Institute. SPF BALB/c mice were infected with tachyzoites (10 mice per strain). Mice were monitored twice daily and euthanized when they exhibited clinical signs indicative of *T. gondii* infection, such as reduced appetite, ruffled fur and head tilting. The body cavity of mice was flushed with sterile phosphate-buffered saline (PBS) and the peritoneal liquid containing the tachyzoites was pelleted by centrifugation at 1,680 × *g* for 15 min. The pellet was washed three times with PBS and the supernatant was discarded after the final wash. Finally, the parasite pellet was resuspended in 1 ml PBS and kept in an Eppendorf tube at −80°C, until use. For collection of tachyzoites of type II PRU *T. gondii*, SPF BALB/c mice were treated with 0.2 mg of dexamethasone (DSMS) in drinking water on alternate days for three times. Then, 10 mice were infected orally with ~150 cysts of *T. gondii* type II PRU strain. Administration of DSMS on alternated days was continued after infection until mice exhibited clinical signs indicative of *T. gondii* infection at 9 days after infection, then mice were sacrificed and tachyzoites of the PRU strain were harvested from the peritoneal cavity of mice as stated above.

### Protein Extraction and Digestion

Protein extraction was obtained from two biological repeats from *T. gondii* tachyzoites of each of the Type I (RH), Type II (PRU) and Chinese 1 (PYS) strains. Briefly, ~5 × 10^8^ tachyzoites of each strain were lysed in a protein lysis buffer (7 M urea/2 M thiourea, 0.2% SDS, 20 mM Tris-HCl, 10 mM DTT, pH 8.5) containing PMSF (1 mM, Thermo Scientific) and phosphatase inhibitors (PhosSTOP, Roche) by sonication (2 s ON/3 s OFF pulses for 5 min on ice). Lysate debris were eliminated by centrifugation at 25,000 *g* for 20 min at 4°C. After centrifugation, samples were deoxidized using 10 mM dithiothreitol (DTT, 60 min, 56°C) and alkylated by 55 mM IAM (45 min at ambient condition). Subsequently, the concentration of protein was determined using the Bradford assay. Protein of each sample (300 μg) were digested by trypsin (Promega) with an enzyme-to-protein ratio of 1:40 overnight at 37°C. Samples were treated with 0.5% (v/v) formic acid (FA) to terminate the enzymatic reaction and then were desalted utilizing Strata X solid-phase extraction columns (Phenomenex). Finally, samples were dried using a SpeedVac and used for iTRAQ labeling.

### iTRAQ Labeling

Samples were labeled with 8-plexed iTRAQ reagent (Applied Biosystems/MDS Sciex, Foster City, CA) according to the manufacturer's recommendations. The label reagents were blended with 50 μl isopropyl alcohol and peptides were resuspended in 30 μl dissolution buffer. Then, label reagents were added into peptides and incubated for 2 h at ambient condition. Labeled peptides were desalted and concentrated on Sep-Pack C_18_ Cartridges (Waters). Proteins of *T. gondii* strains were labeled with iTRAQ tags as follows: Type I RH strain (113 and 114), Chinese 1 PYS strain (115 and 116), and Type II PRU strain (117 and 118).

### Enrichment of Phosphopeptides Using TiO_2_ Beads

The labeled phosphopeptides were enriched by Titanium dioxide (TiO_2_, GL Sciences, Tokyo, Japan). The TiO_2_ beads were suspended in 1 ml loading buffer (2% glutamic acid/65% ACN/2% TFA/) for 10 min. Then, iTRAQ-labeled peptides were added into the freshly prepared TiO_2_ beads at peptides-to-beads ratio of 1:4 (mass/mass). The peptide-beads slurry was shaken on a rotator at 37°C. After 1 h, the blend was pelleted at 12,000 *g* for 5 min, and the supernatant was discarded. The pellet was rinsed three times in 2 ml loading buffer by centrifugation at 12,000 *g* for 5 min. The pellet of the final wash was suspended in 600 μl elution buffer and were shaken for 20 min. Then, the phosphopeptides combined to TiO_2_ were eluted with 500 μl elution buffer, vacuum-dried and subjected to LC-MS/MS analysis.

### LC-MS/MS

LC-MS/MS analysis was performed with the Q Exactive Mass Spectrometer (Thermal Scientific) combined with HPLC (Phenomenex columns Gemini-NX 3u C18 110A 150^*^2.00 mm). The fractionation of the samples was performed using a High pH Reversed-Phase Peptide Fractionation Kit (Thermo Scientific Pierce) according to the manufacturer's instructions. Briefly, the enriched peptides were redissolved with 300 μl 0.1% TFA and then were loaded into the equilibrated fractionation spin column. Peptides were bound to the resin and desalted by washing the column with water using low-speed centrifugation. The volatile high-pH elution with a step gradient of increasing acetonitrile concentrations were used to elute bound peptides from the spin columns into six different fractions. Each fraction was collected and vacuum-dried by centrifugation (Thermo Scientific). Then, samples were dissolved with 20 μl buffer A (2% ACN, 0.1% FA) and were loaded into a C_18_ trap column with a speed of 8 μl/min for 4 min. The linear gradient of buffer B (95% ACN, 0.1% FA) running at 300 nl/min was applied to elute the samples. The elution started from 5% buffer B and increased into 35% buffer B. Then, the concentration of buffer B gradually rose into 60% within 5 min and the linearizing gradient of buffer B ascending into 80% for 2 min was followed. Furthermore, the concentration of buffer B was held at 80% for 2 min and was returned into 5% within 1 min, followed by a maintenance of 5% buffer B for 10 min. Finally, the spectra of first-grade MS were acquired during the scan scope of 350–1,800 m/z in the resolution of 70,000. The AGC (automatic gain control) target and maximum injection time of the first-grade MS were 3e6 and 40 ms, respectively. The spectra of second-grade MS were obtained using the following parameter: resolution = 17,500; maximum injection time = 40 ms; AGC target = 1e5. The mode of second-grade MS spectra was HCD (high-energy collisional dissociation) and the normalized collision energy of which was set as 30 eV.

### Data Analysis

Database search was performed using Proteome Discoverer 1.4 (Thermo Fisher Scientific) implementing a Mascot search engine (version 2.3, Matrix Science). Parameters of the search included: peptide mass tolerance = 20 ppm; enzyme = trypsin; fragment mass tolerance = 0.05 Da; fixed modification = iTRAQ8plex (N-term), carbamidomethyl (C), iTRAQ 8plex(K); variable modification = oxidation (M), acetyl (protein N-term), deamidated (NQ); phosphorylation (S/T/Y) max missed cleavage = 2. The *P*-value for peptide identification and quantification was set as *P* ≤ 0.05. Phosphopeptides with |log1.5-fold change| > 1 were determined as differentially expressed phosphopeptides (DEPSs). The DEPs were grouped using hierarchical clustering method and the software Mev (Multiple Experiment Viewer, version 4.9.0) was applied to create the heat maps.

### Bioinformatics

To predict the probable kinase phosphorylation motifs of phosphopeptides identified in our study, the bias of amino acid residues approximating phosphorylation sites was investigated using the online software Motif-X (http://motif-x.med.harvard.edu/). After removal of uncertain phosphorylation sites, phosphopeptides with the length of ±7 aa from the phosphorylation site position were submitted to the Motif-X software. Each motif detected in the alignment was graphically displayed with the logo-like representation. Gene Ontology (GO) analysis (http://www.geneontology.org) was performed to identify functional categories (molecular function, biological process and cell component) of the DEPs. KEGG (Kyoto Encyclopedia of Genes and Genomes) analysis (http://www.genome.jp/kegg/) was carried out to annotate identified DEPs to KEGG pathways. STRING (Search Tool for the Retrieval of Interacting Genes/Protein, http://string-db.org/) database was searched to identify processes that are regulated by phosphorylation and to analyze interaction networks of the DEPs involved. Interactors with a high confidence (combined score ≥ 0.7) were presented in protein–protein interaction (PPI) networks visualized by Cytoscape (version 3.0.2).

### Correlation of *T. gondii* Kinase Peptides With Phosphopeptides

We used HMMER (http://hmmer.org) to search thePfam 31.0 database to identify sequences of the phosphorylated proteins with Pfam matches (Finn et al., 2016). We obtained a list of phosphorylated peptides related to protein kinase and protein tyrosine kinase domains. Then, we performed a Pearson correlational analysis to associate each of the kinase-related phosphorylated peptide with all phosphorylated peptides in the dataset. This analysis was performed using the DEPs ratio values per each *T. gondii* strain. Only correlations >0.998 were considered. Nodes with log_2_ fold change values −0.75 ≥ log_2_ RS1 ≤ 1.50, −0.75 ≥ log_2_ RS2 ≤ 1.00, and −1.00 ≥ log_2_ RS3 ≤ 1.00 were excluded. This network was visualized using Cytoscape 3.6.0 (https://cytoscape.org).

### Prediction of Phospho-Enabled and Phospho-Disabled Protein Interactions

To identify potential interactions that are enabled or disabled by phosphorylation of the specific sites we used Mechismo (Betts et al., 2015). Because Mechismo does not support *T. gondii* proteomes, we used the eggNOG-mapper ([PMC4702882] http://eggnogdb.embl.de/#/app/emapper) to retrieve the human and yeast orthologs of the proteins identified by our phosphoproteomic analysis of *T. gondii* strains. We first extracted the relevant Orthologous Groups (OG), and then searched for the yeast and human orthologs within these groups using BioPython tools (http://biopython.org/). We then used Clustal Omega (Sievers et al., 2011) to align each orthologous sequence to the corresponding *T. gondii* protein, and identified the corresponding phosphorylation sites in the human or yeast proteins. These were used as input to Mechismo (http://mechismo.russelllab.org/) to identify orthologous that enabled or disabled interactions in the human and yeast network. These were then mapped back to *T. gondii* proteome.

## Results

### Identification and Quantification of Phosphopeptides

**Figure 1:**
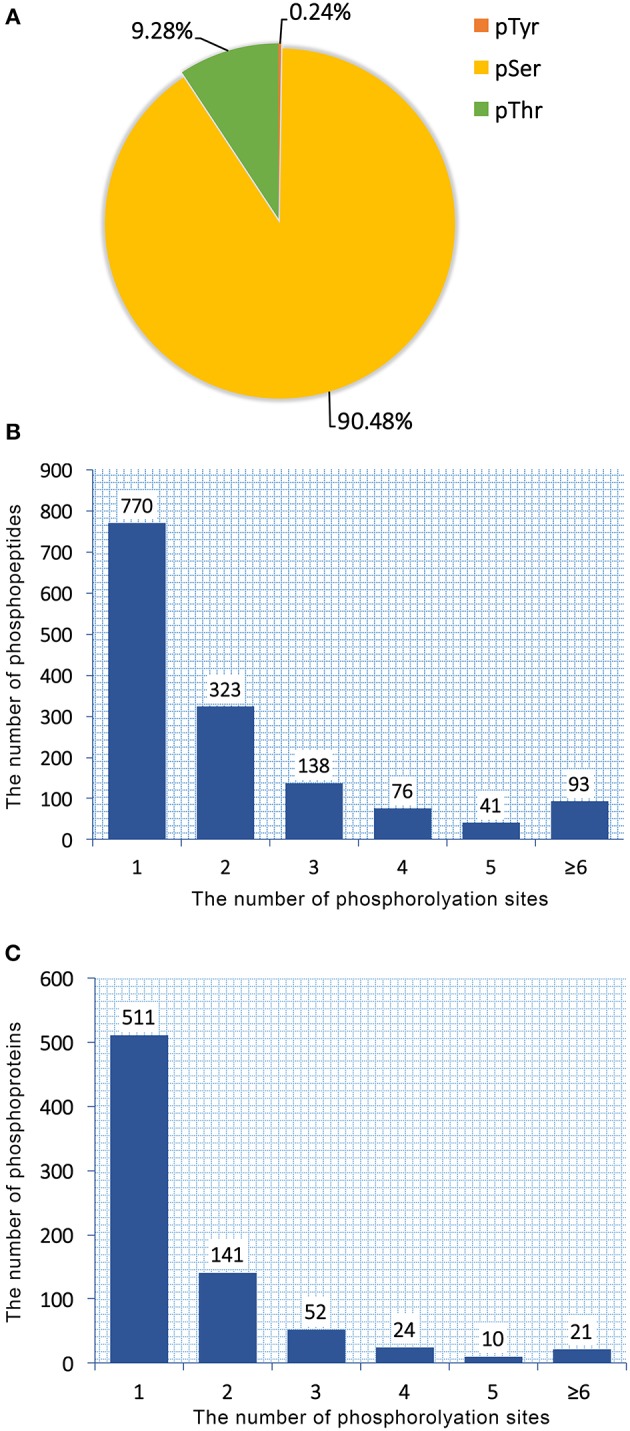
Overall characteristics of the phosphoproteome of RH, PRU and PYS strains of *Toxoplasma gondii*. Distribution of phosphorylated residues and phosphorylation sites per peptide. **(A)** Distribution of phosphorylation on serine (pSer), threonine (pThr), and tyrosine (pTyr) for all phosphorylation sites. **(B)** Number of identified singly and multiply phosphopeptides. The majority of phosphopeptides have only one phosphorylation site. **(C)** Distribution of 759 phosphoproteins based on identification of single or multiple phosphosites per protein. The number of proteins with multiple phosphosites decreases with increasing the number of phosphosites.

Total proteins obtained from *T. gondii* tachyzoites of RH, PRU, and PYS strains were digested and phosphopeptides were enriched by TiO_2_ (titanium dioxide). The enriched fractions were analyzed using LC-MS/MS. A total of 1,441 unique phosphopeptides, 1,250 non-redundant phosphorylation sites and 759 phosphoproteins were detected from *T. gondii* tachyzoites of all three strains (RH, PRU, and PYS) with FDR (false-discovery rate) ≤ 0.01 in phosphopeptide level and phosphoRS probability ≥ 0.75 in phosphorylation site level. The identified 1,250 phosphorylation sites consisted of 1,131 (90.48%) serine phosphorylation (*pSer*), 116 (9.28%) threonine phosphorylation (*pThr*), and 3 (0.24%) tyrosine phosphorylation (*pTyr*) (Figure 1A). Out of the 1,441 phosphopeptides, 770, 323, 138, 76, 41, and 93 were found phosphorylated at 1, 2, 3, 4, 5, and ≥6 phosphorylation sites, respectively (Figure 1B).

**Figure 2:**
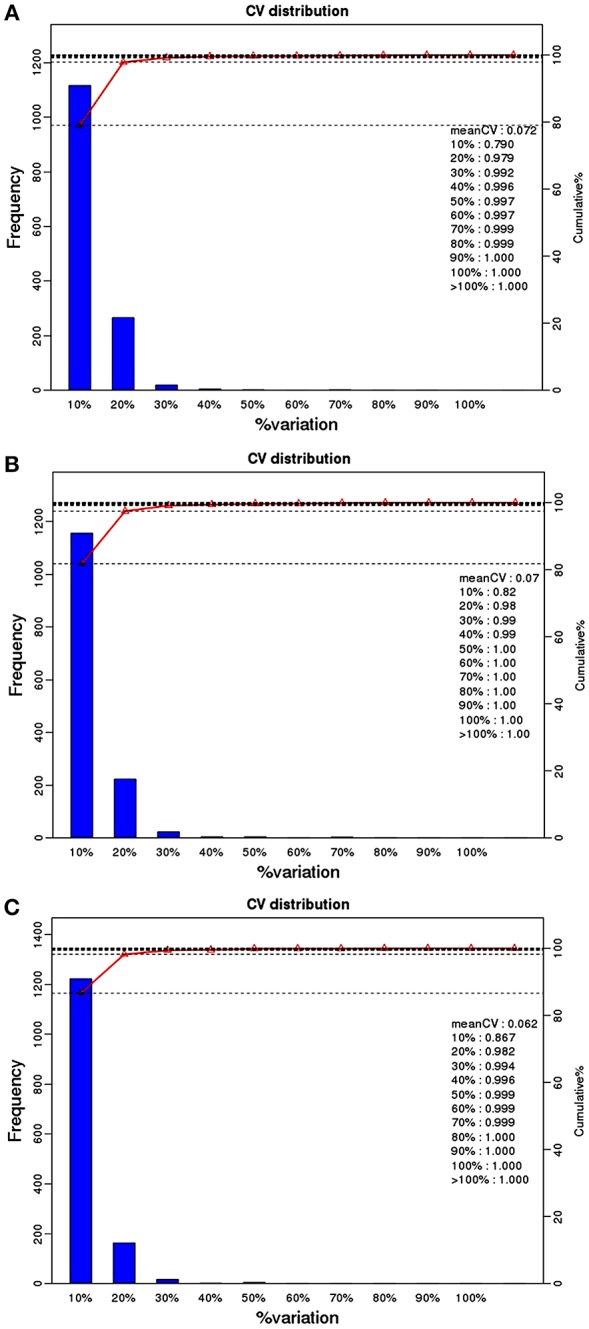
Repeatability assessment of phosphopeptides quantification based on Coefficient of variation (CV) in RH vs. PRU, PRU vs. PYS, PYS vs. RH. The percentage on X-axis represents the values of CV. The left y-axis indicates the number of phosphopeptides and the right y-axis indicates the cumulative percentage of phosphopeptides. **(A–C)** Represent repeatability assessment of RH vs. PRU, PRU vs. PYS, PYS vs. RH, respectively.

759 phosphoproteins were identified, including 511, 141, 52, 24, 10, and 21 phosphoproteins phosphorylated at 1, 2, 3, 4, 5, and ≥6 phosphorylation sites, respectively (Figure 1C). Repeatability analysis represented by the CV (Coefficient of variation) identified 97.9, 98.0, and 98.2% phosphoproteins among total phosphoproteins in RH strain when comparing RH/PRU strains, in PRU strain when comparing PRU/PYS strains, and in PYS strain when comparing PYS/RH strains, respectively, when the CV was set as 0.2 (Figures 2A–C).

### Differentially Expressed Phosphoproteins and Clustering Analysis

**Figure 3:**
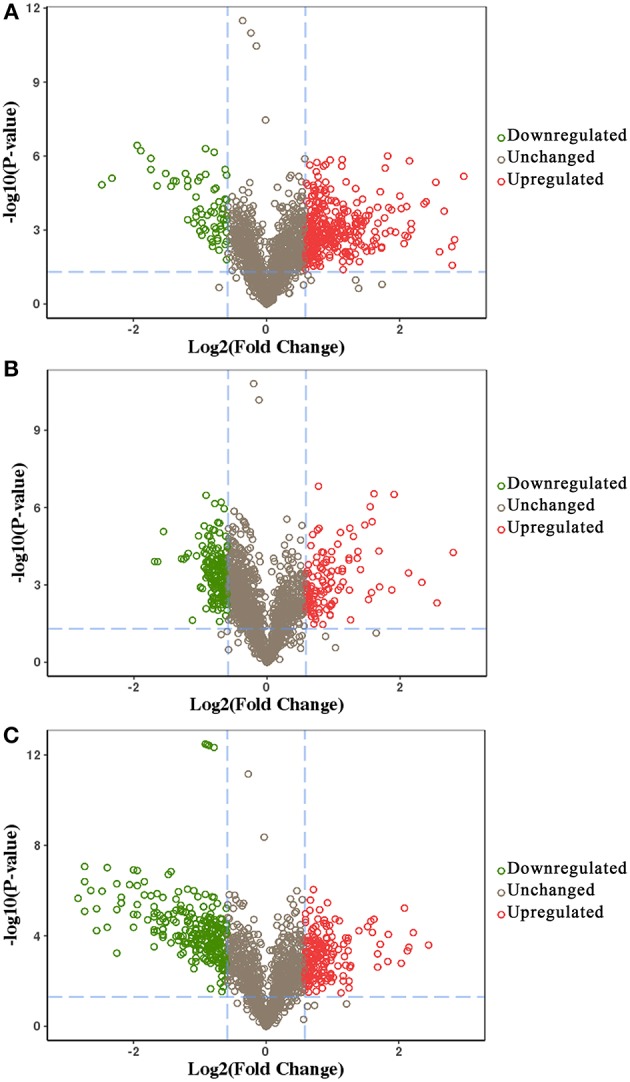
Volcano plots showing the median phosphopeptides log_2_ fold change plotted against the –log_10_ *P*-value. The results highlight the phosphopeptides up/down-regulated in RH strain when comparing RH/PRU strains **(A)**, in PRU when comparing PRU/PYS strains **(B)**, and in PYS strain when comparing PYS/RH strains **(C)**, respectively. Red and green circles represent upregulated and downregulated DEPs, respectively. Dotted lines indicate (±) 1.5-fold change.

**Figure 4:**
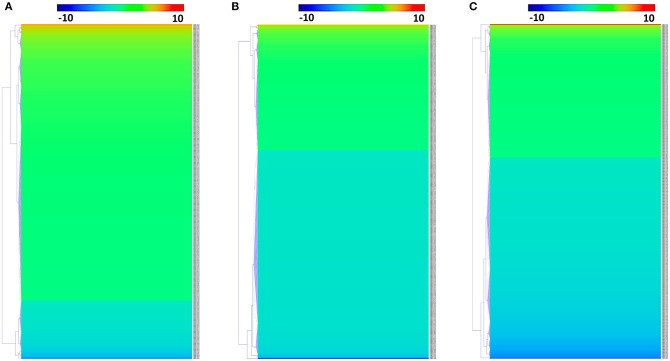
Hierarchical clustering heat maps of differentially expressed phosphoproteins (DEPs). Expression values of DEPs were log1.5-transformed. **(A–C)** Represent heat maps of phosphoproteins up/down-regulated in RH strain when comparing RH/PRU strains, in PRU strain when comparing PRU/PYS strains, and in PYS strain when comparing PYS/RH strains, respectively.

We identified 392, 298, and 436 differentially expressed phosphoproteins (DEPs) in RH strain when comparing RH/PRU strains, in PRU strain when comparing PRU/PYS strains, and in PYS strain when comparing PYS/RH strains, respectively (|log1.5-fold change| > 1 and *p*-value < 0.05) (Tables S1–S3). Among the DEPs, 324 upregulated and 68 downregulated phosphoproteins are found in RH strain when comparing RH/PRU strains (Figure 3A). There are 112 upregulated phosphoproteins and 186 downregulated phosphoproteins in PRU strain when comparing PRU/PYS strains (Figure 3B). Additionally, 174 upregulated phosphoproteins and 262 downregulated phosphoproteins are detected in PYS strain when comparing PYS/RH strains (Figure 3C). Figures 4A–C show the results of the hierarchical clustering analysis of DEPs between the parasite strains (RH vs. PRU, PRU vs. PYS, PYS vs. RH).

### Prediction of p-Site Motifs Using Motif-x

**Figure 5:**
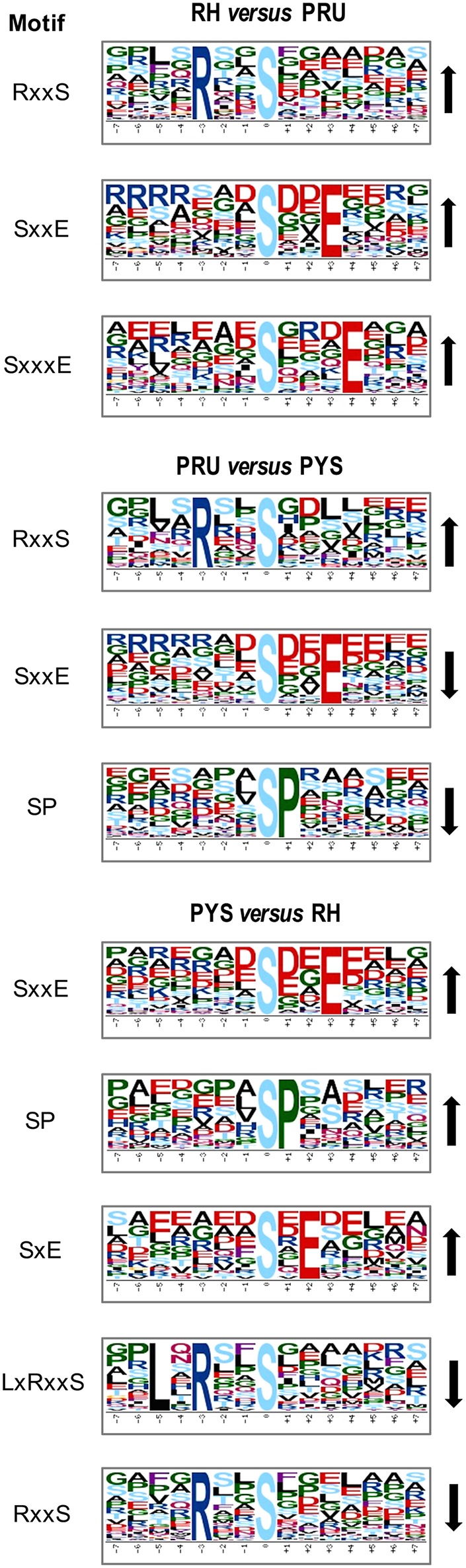
Phosphorylation-specific motif analysis using the Motif-X algorithm. The representations of the serine phosphorylation motifs identified from all unambiguous phosphorylation sites are depicted as sequence logos. The height of the residues represents the frequency with which they occur at the respective positions. The color of the residues represents their physicochemical properties. Arrows pointing up or down on the right of each motif denotes that the motif was up-regulated or down-regulated, respectively.

To evaluate the conservation of sequence surrounding phosphorylation sites, motif analysis was performed between −7 and +7 positions of phosphorylation sites in all identified phosphoproteins and DEPs of RH vs. PRU, PRU vs. PYS, PYS vs. RH to detect the frequencies of amino acid residue at specific positions and identify conserved motifs. This analysis provided insights into classes of kinases differentially activated between tachyzoites of the three *T. gondii* strains. This analysis discovered 17 motifs in all phosphorylated proteins (Table 1) and 11 of these represent motifs of the DEPs between parasite strains (Figure 5). Kinases corresponding to each motif are shown in Table 2. Comparing RH to PRU, three motifs (RxxS, SxxE, and SxxxE) were identified in the up-regulated phosphorylation sites and no motif was detected in the down-regulated phosphorylation sites (Figure 5A). One RxxS motif was found among the up-regulated phosphorylation sites and two motifs (SxxE, SP) were detected among the down-regulated phosphorylation sites in PRU strain when comparing PRU/PYS strains (Figure 5B). For PYS vs. RH, three motifs (SxxE, SP, and SxE) had higher expression level in PYS strain and two motifs (LxRxxS and RxxS) had higher expression level in RH strain (Figure 5C).

**Table 1:** The description of the phosphorylation motifs identified by Motif X analysis.

No.^*^	**Motif^†^**	**Motif score**	**Foreground**		**Background**		**Fold increase**
			**Matches**	**Size**	**Matches**	**Size**	
1.	.R.S.D…. 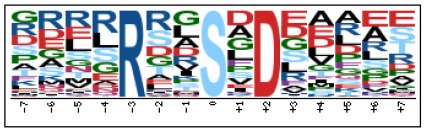	26.42	44	1,080	2,478	715,183	11.76
2.	…….SPR…. 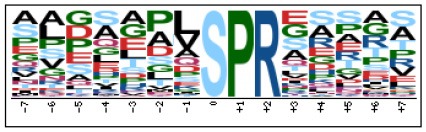	30.84	62	1,036	4,743	712,705	8.99
3.	….R.S.G…. 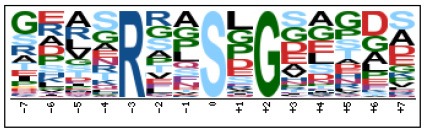	23.82	50	974	4,147	707,962	8.76
4.	….R.SP…… 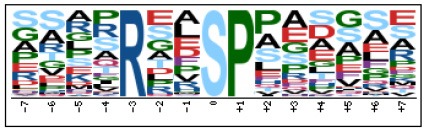	22.98	42	924	3,846	703,815	8.32
5.	….R.S.E…. 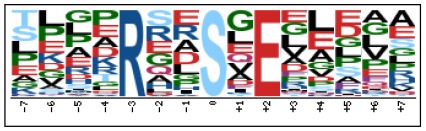	22.95	38	882	3,781	699,969	7.98
6.	…….SD.E…. 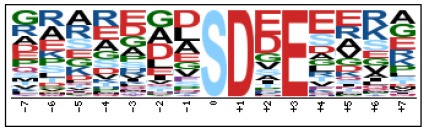	32.00	61	844	2,551	696,188	19.72
7.	….G.SP…… 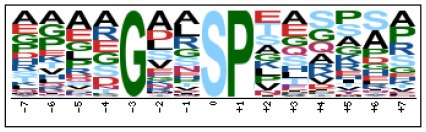	31.95	45	783	3,680	693,637	10.83
8.	….R.S……. 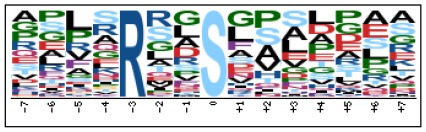	16.00	139	738	41,026	689,957	3.17
9.	…….SP…… 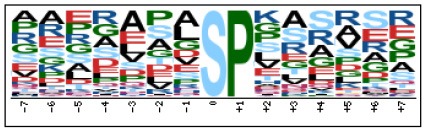	16.00	120	599	52,860	648,931	2.46
10.	…….S.E…. 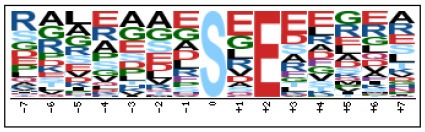	16.00	91	479	35,368	596,071	3.20
11.	…….SD…… 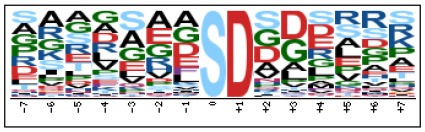	16.00	58	388	20,383	560,703	4.11
12.	…….S.E…. 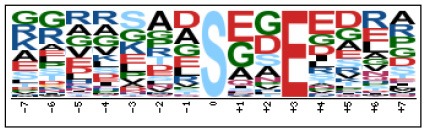	13.68	58	330	30,604	540,320	3.10
13.	……GS……. 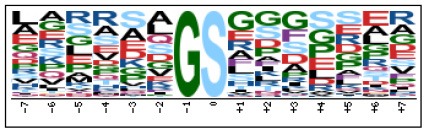	12.68	53	272	32,034	509,716	3.10
14.	….H.S……. 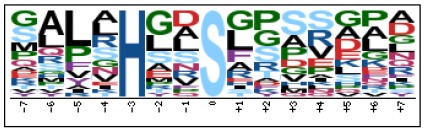	8.09	22	219	10,813	477,682	4.44
15.	…….S.G…. 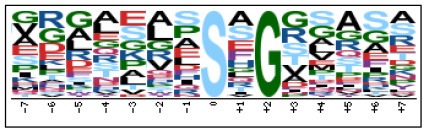	6.69	35	197	31,938	466,869	2.60
16.	……DS……. 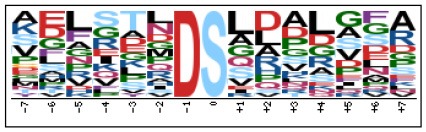	6.49	23	162	18,142	434,931	3.40
17.	…….TP…… 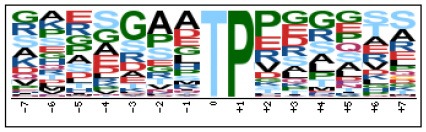	16.00	45	112	24,257	326,403	5.41

* No. 1–16 represent the serine phosphorylation motifs; No. 17 represents the threonine phosphorylation motif; phosphorylation sites were determined by Motif-X.

† *The height of each amino acid represents the frequency of this residue occurring at the position of pS/pT*.

**Table 2:** Types of kinases represented by the 17 motifs listed in Table 1 as determined by KinasePho.

**Motif**	**Kinase classes**
R.S.D	PKA, PKB, PKG, CKI, CKII, IKK, CaM-II, ATM, CDC2
SPR	PKB, IKK, ATM, CDC2, CDK, MAPK
R.S.G	PKA, PKB, PKC, PKG, CKI, CaM-II, IKK, ATM, CDC2
R.SP	PKA, PKB, PKG, CaM-II, IKK, ATM, CDC2, CDK, MAPK
R.S.E	PKA, PKB, PKG, CKI, CKII, IKK, ATM
SD.E	PKG, CKI, CKII, ATM
G.SP	IKK, ATM, CDC2, CDK, MAPK
R.S	PKA, PKB, PKC, PKG, CKI, CKII, IKK, CaM-II, ATM
SP	PKB, PKG, IKK, ATM, CDC2, CDK, MAPK
S.E	PKG, CKI, CKII, ATM
SD	PKA, PKG, CKI, CKII, ATM
S.E	PKA, PKG, CKI, CKII, IKK, ATM
GS	PKA, PKG, CKII, IKK, CaM-II, ATM
H.S	PKA, CKI, ATM, CDC2, MAPK,
S.G	PKC, PKG, CKI, CKII, IKK, ATM, CDC2
DS	CKI, CKII, ATM
TP	PKA, CDC2, CDK, MAPK

*PKA, cAMP-dependent protein kinase; PKB, protein kinase B; PKC, protein kinase C; PKG, cGMP-dependent protein kinase; CKI, casein kinase I; CKII, casein kinase II; IKK, IkappaB kinase; CaM-II, calmodulin-dependent protein kinase II; ATM, ataxia telangiectasia mutated kinase; CDC2, cell division cycle protein kinase p34; CDK, cyclin-dependent kinase; MAPK, mitogen-activated protein kinase*.

### Functional Classification of DEPs Using GO Analysis

**Figure 6:**
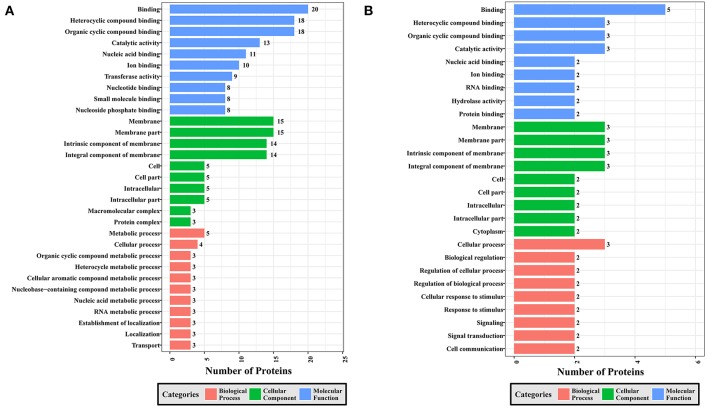
GO analysis of phosphoproteins up/down-regulated in RH strain when comparing RH/PRU strains. The X-axis indicates the number of DEPs. The Y-axis represents GO terms of **(A)** upregulated and **(B)** downregulated phosphoproteins in RH strain when comparing RH/PRU strains, respectively.

**Figure 7:**
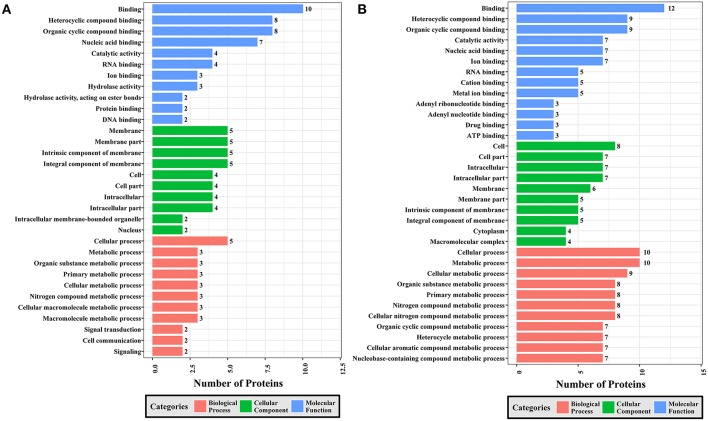
GO analysis of phosphoproteins up/down-regulated in PRU strain when comparing PRU/PYS strains. The X-axis indicates the number of DEPs. The Y-axis represents GO terms of **(A)** upregulated and **(B)** downregulated phosphoproteins in PRU strain when comparing PRU/PYS strains, respectively.

**Figure 8:**
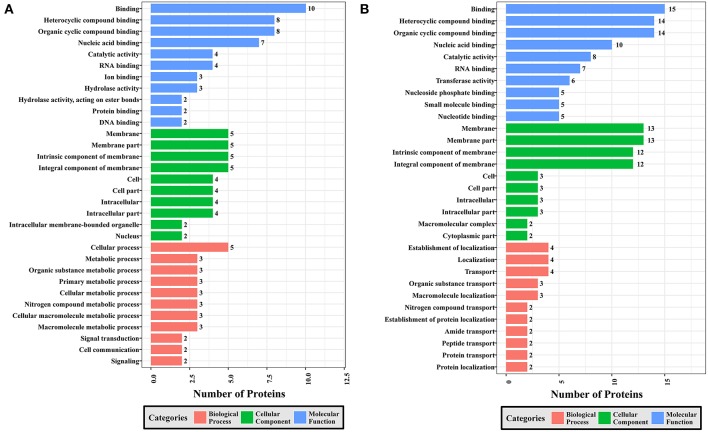
GO analysis of phosphoproteins up/down-regulated in PYS strain when comparing PYS/RH strains. The X-axis indicates the number of DEPs. The Y-axis represents GO terms of **(A)** upregulated and **(B)** downregulated phosphoproteins in PYS strain when comparing PYS/RH strains, respectively.

To gain a better understanding of the functional extent of the differences in protein phosphorylation between strains and its effects on cellular processes, we performed GO enrichment classification of the differentially expressed phosphorylated proteins (Figures 6–8). Comparing RH to PRU, 86, 185, and 30 GO terms were assigned to molecular function (MF), cellular component (CC), and biological process (BP), respectively. The classification of the MF revealed a significant enrichment of both upregulated and downregulated phosphoproteins involved in binding, heterocyclic compound binding and organic cyclic compound binding. In the CC category, significantly enriched upregulated and downregulated phosphoproteins were involved in membrane, membrane part, intrinsic component of membrane, and integral component of membrane. Interestingly, in the BP category, enriched upregulated phosphoproteins were involved in metabolic process, cellular process and organic cyclic compound metabolic process, whereas enriched downregulated phosphoproteins were involved in cellular process, biological regulation, and regulation of cellular process (Figure 6).

In regards to PRU vs. PYS, 69 MF, 130 BP, and 26 CC GO terms were identified. Binding, heterocyclic compound binding and organic cyclic compound binding were the top three MF GO terms, which included most enriched upregulated and downregulated phosphoproteins. In the CC category, the GO term with most enriched downregulated phosphoproteins were cell, cell part, intracellular and intracellular part, whereas the GO term with most enriched upregulated phosphoproteins included membrane, membrane part, intrinsic component of membrane and integral component of membrane. In the BP category, the top three GO terms with most enriched upregulated phosphoproteins were cellular process, metabolic process and organic substance metabolic process. In contract, GO terms cellular process, metabolic process and cellular metabolic process represented the most enriched downregulated phosphoproteins (Figure 7).

Comparing PYS to RH revealed 95 MF, 219 BP, and 46 CC GO terms. The most three abundant GO terms for upregulated or downregulated phosphoproteins under MF category were binding, followed by heterocyclic compound binding and organic cyclic compound binding. For the CC category, membrane, membrane part and intrinsic component of membrane were the mostly prevalent terms for up- and down-regulated phosphoproteins. In the BP category, the most prominent GO terms for upregulated and downregulated phosphoproteins were cellular process and establishment of localization, respectively (Figure 8).

We mapped the extent of association among GO functions by generating co-expression networks of GO terms related to the three functional categories using the R Igraph package. As shown in Figure S1, nine GO terms (heterocyclic compound binding, small molecular binding, organic cyclic compound binding, nucleoside phosphate binding, nucleotide binding, drug binding, ATP binding, adenyl nucleotide binding, and adenyl ribonucleotide binding) under MF category, two terms (membrane and membrane part) under CC category and three terms (localization, establishment of localization and transport) under BP category were significantly enriched in the GO network of upregulated phosphoproteins in RH strain when comparing RH/PRU strains. There were seven, two and three connections among the nine GO terms under MF, two GO terms under CC and three GO terms under BP, respectively. However, only three terms (cell communication, signal transduction and signaling) under BP and no term under MF or CC were significantly enriched in the GO network of downregulated phosphoproteins in RH strain when comparing RH/PRU strains (Figure S2).

There was no significantly enriched GO term in any category of the GO network of upregulated phosphoproteins in PRU strain when comparing PRU/PYS strains (Figure S3). Whereas, each category has significantly enriched GO terms in the GO network of downregulated phosphoproteins in PRU strain when comparing PRU/PYS strains (Figure S4). These included: two GO terms (cation binding and metal ion binding) in the MF category, two terms (nuclear part and nucleoplasm part) in the CC category and 14 enriched terms under the BP category (cellular aromatic compound metabolic process, organic cyclic compound metabolic process, heterocycle metabolic process, nucleic acid metabolic process, nucleobase-containing compound metabolic process, RNA metabolic process, cellular metabolic process, cellular process, cellular macromolecule metabolic process, macromolecule metabolic process, metabolic process, nitrogen compound metabolic process, cellular nitrogen compound metabolic process, and gene expression).

In the GO network of upregulated phosphoproteins in PYS strain when comparing PYS/RH strains, three GO terms (antioxidant activity, peroxidase activity, and oxidoreductase activity, acting on peroxide as acceptor) under MF category were enriched. However, there was no significantly enriched GO term in the CC or the BP category of the GO network of upregulated phosphoproteins (Figure S5). By contrast, the GO network of downregulated phosphoproteins showed two significantly enriched GO terms (heterocyclic compound binding and organic cyclic compound binding) in the MF category and four enriched GO terms (membrane, membrane part, intrinsic component of membrane and integral component of membrane) in the CC category, and 11 GO terms (localization, macromolecule localization, establishment of localization, protein localization, transport, nitrogen compound transport, establishment of protein localization, organic substance transport, protein transport, amide transport, and peptide transport) in the BP category (Figure S6).

### KEGG Pathway Analysis

**Figure 9:**
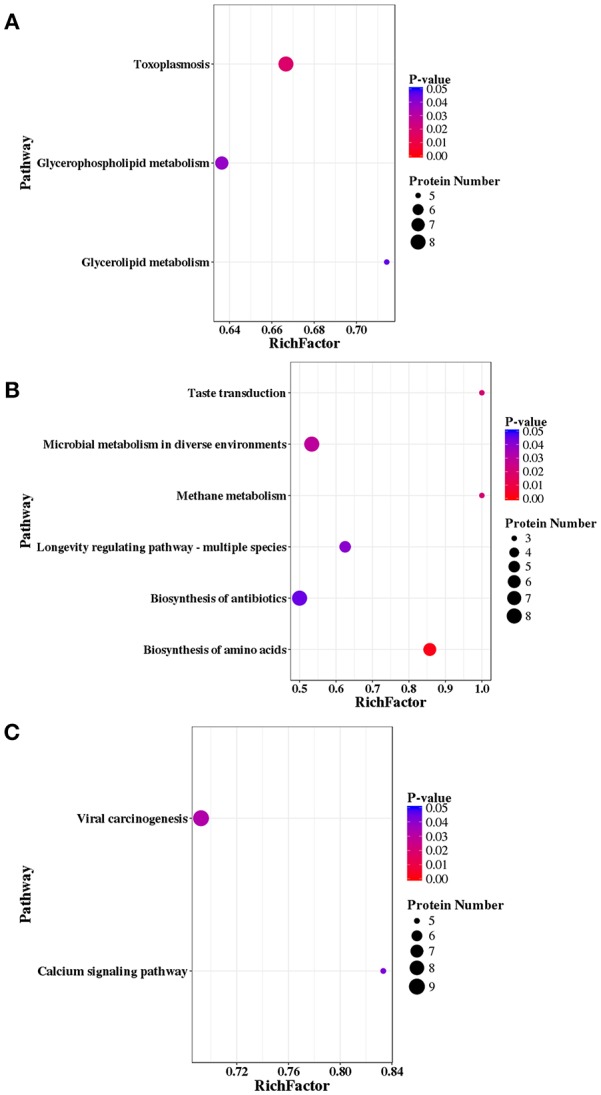
KEGG pathway analysis of differentially expressed phosphoproteins (DEPs) in RH strain when comparing RH/PRU strains, in PRU strain when comparing PRU/PYS strains, and in PYS strain when comparing PYS/RH strains. The vertical axis shows the significantly enriched KEGG pathways and the horizontal axis represents the rich factors corresponding to the pathways. Rich factor refers to the ratio of the number of DEPs to the number of all phosphoproteins in the pathway. Higher Rich factors indicate greater degrees of enrichment. The size and color of the node represent number of phosphoproteins and *p*-value of pathways. The scatter plots represent KEGG pathways of the DEPs in **(A)** RH strain when comparing RH/PRU strains, **(B)** in PRU strain when comparing PRU/PYS strains, and **(C)** in PYS strain when comparing PYS/RH strains, respectively.

A total of 147, 121, and 177 DEPs were annotated in the KEGG database and were mapped to 171, 153, 185 pathways in RH strain when comparing RH/PRU strains, in PRU strain when comparing PRU/PYS strains, and in PYS when comparing PYS/RH strains, respectively. The significantly enriched pathways (*p*-value ≤ 0.05) between the parasite strains are shown in Figure 9. In RH vs. PRU, 8, 7, and 5 phosphoproteins were significantly enriched in toxoplasmosis, glycerophospholipid metabolism, and glycerolipid metabolism pathways, respectively (Figure 9A). In PRU vs. PYS, biosynthesis of amino acids, methane metabolism, taste transduction, microbial metabolism in diverse environments, longevity regulating pathway—multiple species and biosynthesis of antibiotics were the five significantly enriched pathways, and included 6, 3, 3, 8, 5, and 8 phosphoproteins, respectively (Figure 9B). There were two significantly enriched pathways in PYS vs. RH (viral carcinogenesis and calcium signaling pathway), and included 9 and 5 phosphoproteins, respectively (Figure 9C).

To examine the association between enriched pathways, the pathway enrichment network was generated for RH vs. PRU, PRU vs. PYS, and PYS vs. RH. In the context of RH vs. PRU, the significantly enriched pathway “RNA transport” was the most independent, because it was only connected to the “mRNA surveillance pathway.” Four pathways, including “toxoplasmosis,” “AMPK signaling pathway,” “insulin signaling pathway,” and “cGMP-PKG signaling pathway” were linked (Figure S7). The significantly enriched pathway “glycerophospholipid metabolism” was connected with “glycerolipid metabolism,” “phosphonate and phosphinate metabolism,” and “ether lipid metabolism” pathways.

Comparing PRU to PYS, while “RNA transport” pathway was not linked to any other enriched pathways, two significantly enriched pathways “taste transduction” and “tight junction” were connected through the protein kinase (incomplete catalytic triad, a member of protein kinase group AGC) encoded by TGME49_272540 (Figure S8). Other four significantly enriched pathways: “biosynthesis of amino acids,” “biosynthesis of antibiotics,” “microbial metabolism in diverse environments,” and “methane metabolism” were connected by gamma-glutamyl phosphate reductase (TGME49_270550), GAPDH1 (glyceraldehyde-3-phosphate dehydrogenase, TGME49_289690), pyruvate kinase PyK1 (TGME49_256760), transaldolase (TGME49_229360), ATP-citrate lyase (TGME49_223840), fructose-1,6-bisphosphate aldolase (TGME49_236040), phosphoserine aminotransferase (TGME49_218780), and fructose-bisphospatase I (TGME49_205380).

Regarding the network concerning PYS vs. RH, all enriched pathways were directly or indirectly connected with other pathways (Figure S9). The protein kinase (incomplete catalytic triad, a member of protein kinase group AGC) encoded by TGME49_272540 was directly connected to two significantly enriched pathways, namely “viral carcinogenesis” and “calcium signaling pathway.”

### Protein-Protein Interactions (PPI)

**Figure 10:**
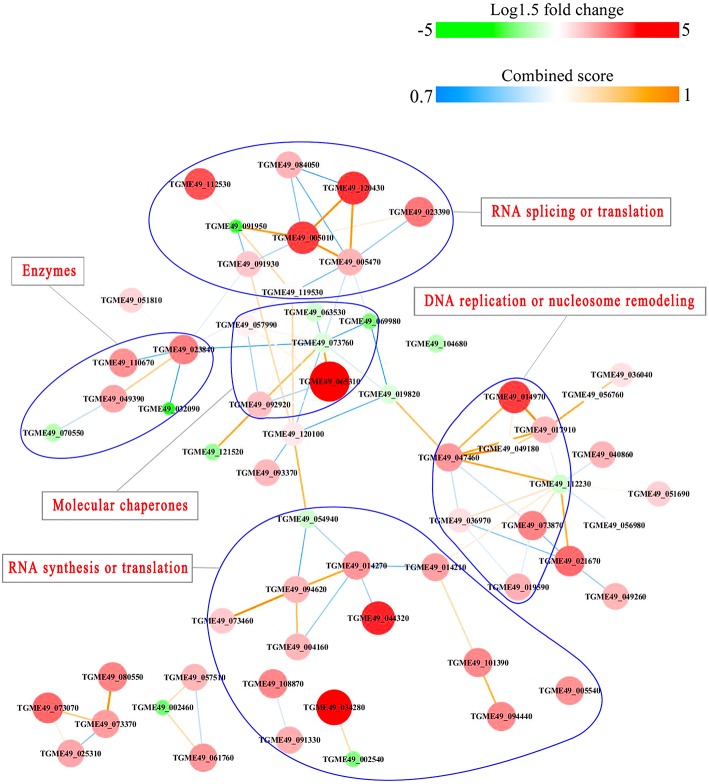
Interaction network (combined score > 0.7) of the identified phosphoproteins up/down-regulated in RH strain when comparing RH/PRU strains. The nodes represent phosphoproteins and the edges denote interactors between phosphoproteins. The upregulation and downregulation of phosphoproteins were characterized by the color and size of nodes. Red and large size nodes denote up-regulated phosphoproteins in RH, whereas green and small size nodes indicate down-regulated phosphoproteins in RH. The color of edge represents the combined score of interactors.

**Figure 11:**
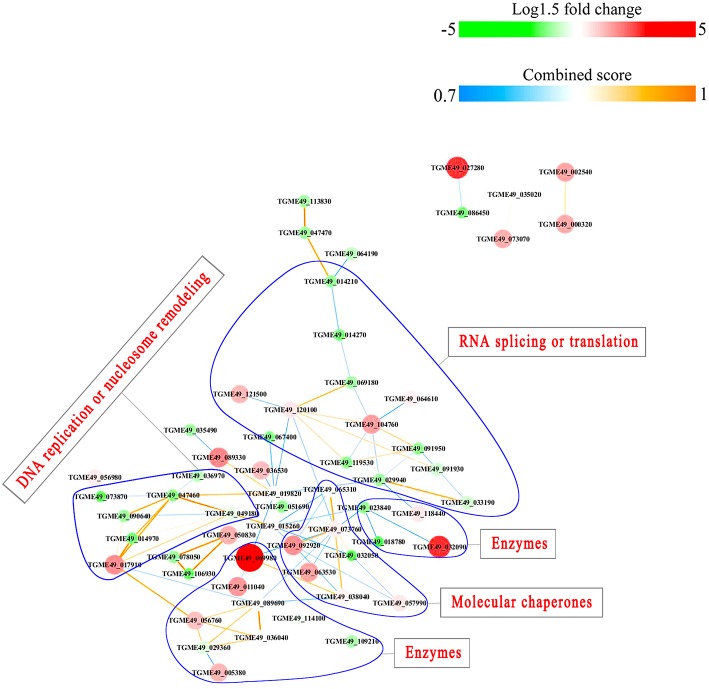
Interaction network of the phosphoproteins up/down-regulated in PRU strain when comparing PRU/PYS strains. Nodes represent phosphoproteins and edges denote interactors between phosphoproteins. The upregulation and downregulation of phosphoproteins were characterized by the color and size of nodes. Red and large size nodes represent up-regulated phosphoproteins in PRU, whereas green and small size nodes denote down-regulated phosphoproteins in PRU. The color of edge represents the combined score of interactors.

**Figure 12:**
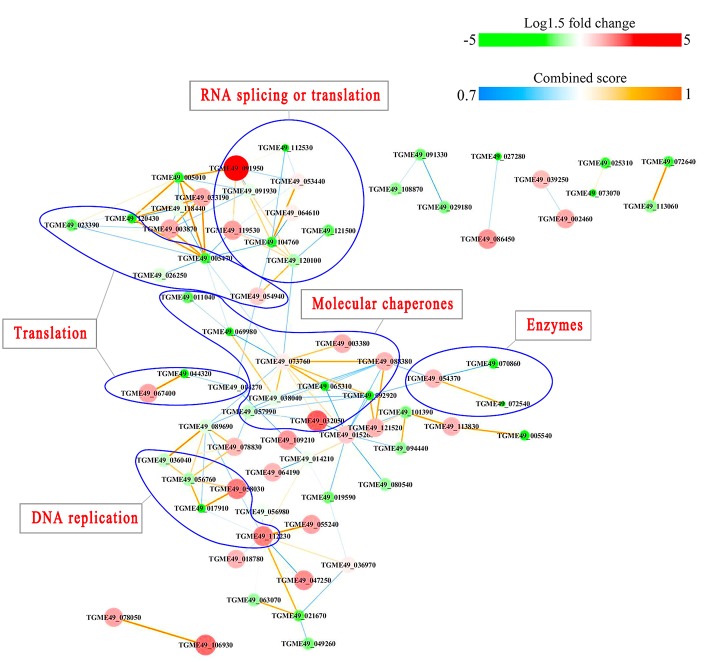
Interactions network of the phosphoproteinsup/down-regulated in PYS strain when comparing PYS/RH strains. Nodes represent phosphoproteins and edges denote interactors between phosphoproteins. The upregulation and downregulation of phosphoproteins were characterized by the color and size of nodes. Red and large size nodes represent up-regulated phosphoproteins in PYS, whereas green and small size node denote down-regulated phosphoproteins in PYS. The color of edge represents the combined score of interactors.

PPI networks (combined score > 0.7) were constructed to understand the regulatory mechanisms of PTMs and to identify the relevant functional clusters of the DEPs between the parasite strains. The PPI network of up/down-regulated phosphoproteins in RH when comparing RH/PRU included 62 protein nodes and 96 interactor edges. Phosphoproteins involved in RNA splicing or translation, DNA replication or nucleosome remodeling, enzymes, molecular chaperones and RNA synthesis or translation formed major hubs (Figure 10). The PPI network of up/down-regulated phosphoproteins in PRU when comparing PRU/PYS involved 59 nodes and 98 edges and contained RNA splicing or translation, DNA replication or nucleosome remodeling, enzymes and molecular chaperones clusters (Figure 11). There were 73 nodes and 133 edges in the PPI network of up/down-regulated phosphoproteins in PYS strain when comparing PYS/PRU strains (Figure 12), which included RNA splicing or translation, DNA replication, enzymes, molecular chaperones, and translation functional clusters.

### Kinase Associated Network

Based on the assumption that phosphorylation affects the kinase activity of the protein, we performed a correlation analysis to identify potential peptides whose phosphorylation was associated with that of the respective kinase (Table S4). We identified 11 phosphorylated kinases, which after keeping phosphopeptides with > ~2-fold change abundance, were found to be correlated with 132 phosphopeptides. Positive correlation indicates that phosphorylation of the kinase has a positive effect on its function leading to downstream effects similar to the kinase phosphorylation status, whereas negative correlation implies the opposite effect. While these correlations cannot confirm a regulatory relationship, they may suggest potential kinases and associated substrates that are different among *T. gondii* strains. Peptides related to *T. gondii* rhoptry protein 16 A0A125YY08 (connected with 84 phosphorylated peptides), cell-cycle-associated protein kinase CDK S8EVL9 (connected with 84 phosphorylated peptides) and type II rhoptry protein 5 A0A125YQ30 (connected with 89 phosphorylated peptides) were the most connected kinase peptides. Kinases S8G171 and S8GM6 formed a second cluster, and the third cluster of phosphorylated peptides encompassed two proteins: cell-cycle-associated protein kinase CDK A0A125YUD2 and S8FAN7 (Figure S10). The most connected kinases peptides (ROP5, ROP16 and cell-cycle-associated protein kinase CDK) were enriched in molecular function GO terms related to ATP binding, cyclin-dependent protein serine/threonine kinase activity, MAP kinase activity, and RNA polymerase II carboxy-terminal domain kinase activity.

### Potential Phosphor-Dependent Interactions

We then sought to identify the putative role of the different phosphorylation sites in our dataset within the context of the *T. gondii* protein interaction network. After mapping our *T. gondii* proteins to their human orthologs and using Mechismo, which predicts whether a mutation or phosphorylation affects the interaction between two proteins by enabling or disabling it, we identified a total of 83 interacting partners of our *T. gondii* orthologs as enabled or disabled by phosphorylation of our identified sites (Table S5). Among these, 16 could be mapped back to *T. gondii* proteins and therefore were identified as putatively affected protein interactions in *T. gondii* (Figure S10). The S700 phosphosite of *T. gondii* S8EUC5 gene affected 47 interactions and phosphosite T1189 of *T. gondii* S8EVL9 gene disabled 8 interactions. On the other hand, the S420 phosphosite of *T. gondii* A0A125YSW4 gene disabled 6 interactions and the S355 phosphosite of S8GUL9 gene enabled 2 interactions and disabled 10 interactions.

## Discussion

Pathogenic *T. gondii* strains seem to rely on the ability to orchestrate the expression of virulence-associated genes (Yang et al., 2013) and proteins (Zhou et al., 2017) for successful infection. Many protein kinases have been also shown to influence the parasite virulence, suggesting a correlation between protein phosphorylation and *T. gondii* virulence. However, knowledge of the phosphoproteomic profile of strains of different virulence capabilities remains limited. Here, we performed a global phosphoproteomic profiling of *T. gondii* strains (type I [RH strain], type II [PRU strain], and Chinese ToxoDB#9 [PYS strain]) using iTRAQ-based MS approach combined with TiO_2_ affinity chromatography. Our data showed that protein phosphorylation is a key regulatory PTM that plays many roles in the pathobiology of *T. gondii*, at the individual protein level and at the level of signaling pathways as described below.

We identified 1,441 phosphopeptides, 1,250 phosphorylation sites and 759 phosphoproteins. The phosphorylation sites included 1,131 (90.48%) serine phosphorylation (pSer), 116 (9.28%) threonine phosphorylation (pThr), and 3 (0.24%) tyrosine phosphorylation (pTyr). These results seem consistent with previous phosphoproteomic studies in eukaryotes which identified serine (Ser), threonine (Thr), and tyrosine (Tyr) as the most common sites for phosphorylation (Pearlman et al., 2011) and reported the relative abundance of serine, threonine and tyrosine phosphorylation as 90, 10, and 0.05% (Olsen et al., 2006; Huttlin et al., 2010). The fact that phosphoserine represents the most abundant type of phosphorylation is anticipated because most *T. gondii* proteins, which play role in the parasite replication, invasion and virulence, contain serine kinase domains. For example, serine rhoptry kinases (ROP5, ROP16 and ROP18) contribute significantly to the differences observed in the virulence between different genotypes (Taylor et al., 2006; Behnke et al., 2011). Also, TgSR3 is a serine-arginine-rich alternative-splicing factor that modulates alternative splicing of over 1,000 *T. gondii* genes (Yeoh et al., 2015). *T. gondii* tachyzoites of the highly virulent strain seem to employ a number of proteases to modify the host cells, and to facilitate their dissemination inside the body of the host (Ramírez-Flores et al., 2019). Inhibition of *T. gondii* serine proteases involved in the processing of MIC proteins and digestion of host proteins suppressed the parasite development and disrupted its secretory pathway (Shaw et al., 2002). Although threonine phosphorylation is less abundant than serine phosphorylation in *T. gondii*, phosphorylation of both serine and threonine seems to be linked. The activity of type 2C serine-threonine phosphatase can significantly affect the growth of *T. gondii* in the host cells (Jan et al., 2009). ROP18, an important virulent factor in *T. gondii*, is also a highly polymorphic serine-threonine kinase (Du et al., 2014). Underrepresentation of tyrosine phosphorylation compared to serine and threonine phosphorylation has been reported earlier in apicomplexan parasites (Treeck et al., 2011). Despite its limited contribution, tyrosine phosphorylation can be involved in important signaling pathways, such as MAPK pathway (Lim and Pawson, 2010; Treeck et al., 2011).

### Motifs

Assigning the sequence of phosphosite motifs to special enzyme families can assign phosphoproteins into kinases. Motif analysis identified three, three, and five motifs from the DEPs in RH strain when comparing RH/PRU strains, in PRU strain when comparing PRU/PYS strains, and in PYS strain when comparing PYS/RH strains, respectively (Figures 5A–C). In RH vs. PRU strain, three motifs (RxxS, SxxE, and SxxxE) were upregulated. In PRU vs. PYS strain, the motif (RxxS) was up-regulated and two motifs (SxxE and SP) were down-regulated. In PYS vs. RH strain, three motifs (SxxE, SP, and SxE) in PYS strain and two motifs (LxRxxS and RxxS) in RH strain were up-regulated. Each motif is consistent with one or several category enzymes. For example, enzymes consistent with motif RxxS include PKA, PKB/AKT, PKC, PKG, CKI, CKII, IKK, CaM-II, and ATM; enzymes consistent with motif SxxE include PKA, PKG, CKI, CKII, IKK, and ATM; and enzymes consistent with motif SP included PKB, PKG, IKK, ATM, CDC2, CDK, and MAPK (Nakayasu et al., 2013). These data show the differences in the kinases expressed in the tachyzoites of the three *T. gondii* strains. Motifs are critical amino acid sequences that play roles in the recognition of kinase to its substrate (Schwartz and Gygi, 2005; Pi et al., 2016). Therefore, these strain-specific differences in motifs reflect differences in the substrate-recognition abilities between *T. gondii* strains of different genotypes.

In *T. gondii*, cAMP-dependent protein kinase A (PKA) and cGMP-dependent protein kinase G (PKG) play a role in the control of parasite egress from host cells (Jia et al., 2017). CK I and CK II are conserved serine/threonine protein kinases, and are involved in DNA repair and actin recruitment in eukaryotes, respectively. The function of CK I in the lifecycle of *T. gondii* has not been determined as yet, however, a casein kinase (CK) II-like activity and parasitic type 2C phosphatase were found to modulate the phosphorylation of Toxofilin protein, which plays a role in the actin sequestration and motility of *T. gondii* (Delorme et al., 2003; Wei et al., 2013). *T. gondii* IκB kinase (IKK) phosphorylates host IκBα localized at the PVM in infected cells and modulation of the NF-κB pathway in host cell (Molestina and Sinai, 2005). The upregulation of RxxS and SxxE motifs in RH strain, and SxxE and SP motifs in PYS strain, compared to PRU strain, suggest that the functions of PKA, PKG, CKII and IKK kinases representing these motifs (e.g., parasite egress, actin dynamic, and manipulation of NF-κB pathway in host cell) are probably more prominent in the virulent RH and PYS strains than in the avirulent PRU strain.

*T. gondii* TPK2, a homolog of the CDC2 cyclin-dependent kinase, plays a role in the regulation of the host cell cycle (Khan et al., 2002). In addition, cyclin-dependent kinase 7 (Cdk7) of *T. gondii* can mediate mRNA synthesis (Deshmukh et al., 2016). The activity of *T. gondii* cyclin-dependent kinase 9 is regulated by Cdk7 (Deshmukh et al., 2018). *T. gondii* has two MAPK isomers, TgMAPK1 and TgMAPK2, which play roles in cellular processes, especially in stress response (Wei et al., 2013). The ability of *T. gondii* to interfere with the cell cycle and adapt to stresses inside the host is crucial for pathogenesis. Given that motif SP was upregulated in PYS strain compared to other strains, it is reasonable to assume that intracellular parasite progression and stress tolerance mediated by CDC2-like TPK2, CDKs, and MAPKs are more prominent in PYS strain; however, this remains to be confirmed.

### Functional Characterization of the Phosphorylated Proteins

Here, all identified phosphoproteins were assigned to GO terms in the MF, BP, and CC categories. The top GO terms in the MF were involved in binding and catalytic activities (Figures 6–8), indicating the critical role of phosphorylation in the parasite motility and host cell invasion. Among all phosphorylated proteins of *T. gondii* strains, the largest percentage of CC terms were involved in the cell, cell part, intracellular, intracellular part, and membrane. Other enriched phosphoproteins were related to intracellular membrane bound organelle, and macromolecular complex and other cellular components, suggesting that *T. gondii* strains are well-equipped to deal with long-term interaction of the surrogate host cell. Notably, we detected a significant enrichment of phosphoproteins in the membrane even without using any enrichment or isolation procedures for this organelle. The signal transduction originates at the membrane, where phosphorylation of signaling proteins is a key step to mount an effective response to a perceived stress and to transmit a certain message. The highly enriched GO terms in the BP were involved in cellular process, primary metabolic process, organic substance metabolic process, and cellular metabolic process, highlighting the correlation between bioenergetics and parasite virulence. The fact that binding and metabolic processes are the most highly enriched GO terms agrees with the previous finding that *T. gondii* aldolase (Starnes et al., 2009) and fructose-1,6-bisphosphate aldolase–adhesin (Shen and Sibley, 2014) play a role in energy production and host cell invasion.

KEGG pathway analysis showed that DEPs were annotated into pathways with direct relevance to *T. gondii* high metabolic and bioenergetics demands required to sustain the parasite growth and replication, such as toxoplasmosis, glycerophospholipid metabolism and glycerolipid metabolism, biosynthesis of amino acids, methane metabolism, microbial metabolism in diverse environments, longevity regulating pathway—multiple species and biosynthesis of antibiotics. However, some DEPs (PKA, CAP-Rf, HAUSP, SKIP, and M2-PK) were annotated into pathways that might seem unrelated to *T. gondii*, such as “viral carcinogenesis” pathway. However, PKA and CAP-Rf was found to participate in the pathway of hepatocellular carcinoma caused by hepatitis C virus, and HAUSP and SKIP were involved in the pathway of lymphoma by Epstein-Barr virus (Saha and Robertson, 2011; Vescovo et al., 2016). Also, M2-PK was involved in pathway of cancer caused by human papillomavirus (He et al., 2003). Given the similarity between *T. gondii* and cancer (Lun et al., 2015) and the growing evidence for the correlation between *T. gondii* infection and cancer (Thirugnanam et al., 2013), the role of these DEPs in *T. gondii* virulence would be interesting to unravel.

Results of PPI network analysis showed that the upregulated phosphoproteins involved in RNA splicing or translation, DNA replication or nucleosome remodeling, molecular chaperones and RNA synthesis or translation in the virulent RH strain, and the upregulated phosphoproteins involved in RNA splicing or translation, molecular chaperones in the virulent PYS strain seem to be overrepresented compared to the avirulent PRU strain. Several epigenetic control mechanisms are responsible for a range of biological processes in the tachyzoite and bradyzoite stages of the life-cycle, from the parasite growth to phenotypic plasticity. For example, TgCARM1, TgGCN5-B, and TgMYST-A and -B are essential histone modifying enzymes that contribute to the propagation of tachyzoites (Dixon et al., 2010). TgSET8 and the phosphorylation of histone H3 on Serine-10 have been implicated in cell cycle regulation of *T. gondii*. KMTox and TgMYST-B have been linked to the parasite's response to reactive oxygen species and DNA damage, respectively. TgSRCAP and TgGCN5-A have been implicated in the differentiation of tachyzoites into bradyzoites. In a sharp contrast, phosphoproteins involved in only enzymatic processes seem to be more abundant in avirulent PRU strain compared to the virulent PYS strain. Parasite enzymes involved in dopamine production (Prandovszky et al., 2011), metabolism of oxygen radicals, glycolysis and DNA repair are upregulated in bradyzoites to support the parasite dormancy (reviewed in Sullivan and Jeffers, 2012). Also, the eIF2 kinases in *T. gondii* phosphorylate TgIF2a to elicit translation control in order to endure stresses that the parasite encounters during its life cycle (Joyce et al., 2011). Additionally, *T. gondii* fructose 1,6-bisphosphatase enzyme is essential in glucose metabolism and plays a role in parasite replication *in vitro* and virulence *in vivo* (Blume et al., 2015). These results demonstrate the correlation between the protein phosphorylation and the potential pathogenicity or latency of *T. gondii* strains.

### Kinase-Protein Interactions

Protein kinases phosphorylate specific substrates to control certain cellular processes. We predicted the potential kinase-protein interactions and identified specific parasite peptides related to the phosphorylated peptides. The parasite kinases (ROP16, ROP5 and cell-cycle-associated protein kinase CDK) had the greatest connected peptides and had regulatory roles in ATP binding, MAP kinase activity, RNA polymerase II carboxy-terminal domain kinase activity, and cyclin-dependent protein serine/threonine kinase activity. These findings show the putative role of phosphoproteins in orchestrating *T. gondii* invasion and metabolic regulation.

## Conclusions

This study profiles the global phosphoproteome of *T. gondii* strains belonging to three different genotypes. Combined iTRAQ and TiO_2_ affinity chromatography analysis revealed marked differences in the phosphorylation patterns of tachyzoites between three *T. gondii* strains, which could be contributing to the differences in their virulence potential. The role of phosphorylation in the alteration of parasite kinases was evident from the kinase associated network analysis, which showed ROP5, ROP16, and cell-cycle-associated protein kinase CDK to be the most connected kinases peptides. Differentially abundant phosphoproteins (DEPs) were significantly enriched in several pathways related to the pathogenesis of *T. gondii*. The relevance of the identified DEPs should be characterized in subsequent studies using targeted quantitative phosphoproteomics tools. Because different strains of *T. gondii* have significantly varied pathogenicity, it will be of interest in the future to interrogate other *T. gondii* strains and genotypes, which could help to explain inter-strain and inter-genotype differences in virulence.

## Data Availability

All the mass spectrometry data have been submitted to the ProteomeXchange Consortium with identifier PXD007777.

## Ethics Statement

All animal experiments were conducted with the approval of the Animal Ethics and Administration Committee of Lanzhou Veterinary Research Institute, Chinese Academy of Agricultural Sciences, and in strict accordance with the Animal Ethics Procedures and Guidelines of the People's Republic of China. Every effort was made to reduce the suffering of the animals used in the experiment.

## Author Contributions

X-QZ and HE conceived and designed the experiments. Z-XW and C-XZ performed the experiments, analyzed the data, and wrote the paper. GC-M, EP, J-JH, and H-YS contributed the reagents materials analysis tools. HE and X-QZ critically revised the manuscript. All authors read and approved the final version of the manuscript.

### Conflict of Interest Statement

The authors declare that the research was conducted in the absence of any commercial or financial relationships that could be construed as a potential conflict of interest.

## Supplementary Material

Figure S1 GO function network of upregulated phosphoproteins in RH strain when comparing RH/PRU strains. The clusters coincide with the GO functional categories of the DEPs and are color-coded as indicated. Red, green and blue clusters denote GO terms related to biological process, cellular component and molecular function, respectively. The dark colored nodes represent significantly enriched GO terms (*p*-value ≤ 0.05). The solid and dashed arrows between nodes represent direct and indirect associations between GO terms, respectively.

Figure S2 GO function network of downregulated phosphoproteins in RH strain when comparing RH/PRU strains. The clusters coincide with the GO functional categories of the DEPs and are color-coded as indicated. Red, green and blue clusters denote GO terms related to biological process, cellular component and molecular function, respectively. The dark colored nodes represent significantly enriched GO terms (*p*-value ≤ 0.05). The solid and dashed arrows between nodes represent direct and indirect associations between GO terms, respectively.

Figure S3 GO function network of upregulated phosphoproteins in PRU strain when comparing PRU/PYS strains. The clusters coincide with the GO functional categories of the DEPs and are color-coded as indicated. Red, green, and blue clusters denote GO terms related to biological process, cellular component and molecular function, respectively. The solid and dashed arrows between nodes represent direct and indirect associations between GO terms, respectively.

Figure S4 GO function network of downregulated phosphoproteins in PRU strain when comparing PRU/PYS strains. The clusters coincide with the GO functional categories of the DEPs and are color-coded as indicated. Red, green, and blue clusters denote GO terms related to biological process, cellular component and molecular function, respectively. The dark colored nodes represent significantly enriched GO terms (*p*-value ≤ 0.05). The solid and dashed arrows between nodes represent direct and indirect associations between GO terms, respectively.

Figure S5 GO-network of upregulated phosphoproteins in PYS strain when comparing PYS/RH strains. Red nodes, green nodes, and blue nodes denote GO terms under biological process, GO terms under cellular component and GO terms under molecular function, respectively. Nodes with deeper red, deeper green, and deeper blue represent significantly enriched GO terms (*p*-value ≤ 0.05) under biological process, cellular component and molecular function, respectively. Edges (solid and dotted arrows between nodes) represent direct (solid lines) and indirect (dashed lines) interactions between molecules.

Figure S6 GO function network of downregulated phosphoproteins in PYS strain when comparing PYS/RH strains. The clusters coincide with the GO functional categories of the DEPs and are color-coded as indicated. Red, green, and blue clusters denote GO terms related to biological process, cellular component and molecular function, respectively. The dark colored nodes represent significantly enriched GO terms (*p*-value ≤ 0.05). The solid and dashed arrows between nodes represent direct and indirect associations between GO terms, respectively.

Figure S7 KEGG network of differentially expressed phosphoproteins in RH strain when comparing RH/PRU strains. Light purple nodes represent non-significantly enriched pathways and deep purple nodes indicate significantly enriched pathways (*p*-value ≤ 0.05). The gray nodes surrounding purple nodes denote differentially expressed phosphoproteins.

Figure S8 KEGG network of differentially expressed phosphoproteins in PRU strain when comparing PRU/PYS strains. Light purple nodes represent non-significantly enriched pathways and deep purple nodes indicate significantly enriched pathways (*p*-value ≤ 0.05). The gray nodes surrounding purple nodes denote differentially expressed phosphoproteins.

Figure S9 KEGG network of differentially expressed phosphoproteins in PYS strain when comparing PYS/RH strains. Light purple nodes represent non-significantly enriched pathways and deeper purple nodes indicated significantly enriched pathways (*p*-value ≤ 0.05). The gray nodes surrounding purple nodes denote differentially expressed phosphoproteins.

Figure S10 Network of kinase-phosphopeptide associations and potential phosphor-dependent interactions identified by correlation analysis. Log_2_ (fold changes) of phosphoproteins up/down-regulated in RH when comparing RH/PRU, PRU when comparing PRU/PYS and PYS when comparing PYS/RH were mapped in the nodes in blue, red, and green, respectively. The gray arrow indicates protein interactions that are predicted to be disabled by the phosphorylation and green arrows indicate protein interactions that are predicted to be enabled by the phosphorylation.

Table S1 List of phosphoproteins up/down-regulated in RH strain when comparing RH/PRU strains (|log1.5-fold change| > 1 and *p*-value < 0.05).

Table S2 List of phosphoproteins up/down-regulated in PRU strain when comparing PRU/PYS strains (|log1.5-fold change| > 1 and *p*-value < 0.05).

Table S3 List of phosphoproteins up/down-regulated in PYS strain when comparing PYS/RH strains (|log1.5-fold change| > 1 and *p*-value < 0.05).

Table S4 Correlation values between kinase-related phosphopeptides and associated phosphopeptides.

Table S5 Interactions of human orthologous proteins affected by phosphorylation detected by Mechismo (http://mechismo.russelllab.org/). The interaction effects involved four categories: enabling, enablingWeak, disabling, and disablingWeak and Interaction Confidences involved three categories: High, Medium, and Low. *T. gondii* proteins and phosphorylation sites measured in our experiment were mapped to their human orthologs and Mechismo was used to predict whether these phosphorylations enable or disable interactions in the human network.

## References

[B1] Amorim J. C. Batista M. Da Cunha E. S. Lucena A. C. R. Lima C. V. P. Sousa K.. (2017). Quantitative proteome and phosphoproteome analyses highlight the adherent population during *Trypanosoma cruzi* metacyclogenesis. Sci. Rep. 7:9899. 10.1038/s41598-017-10292-3 28852088 PMC5574995

[B2] Becker C. H. Bern M. (2011). Recent developments in quantitative proteomics. Mutat. Res. 722, 171–182. 10.1016/j.mrgentox.2010.06.016 20620221 PMC2980806

[B3] Behnke M. S. Khan A. Wootton J. C. Dubey J. P. Tang K. Sibley L. D. (2011). Virulence differences in *Toxoplasma* mediated by amplification of a family of polymorphic pseudokinases. Proc. Natl. Acad. Sci. U.S.A. 108, 9631–9636. 10.1073/pnas.1015338108 21586633 PMC3111276

[B4] Betts M. J. Lu Q. Jiang Y. Drusko A. Wichmann O. Utz M.. (2015). Mechismo: predicting the mechanistic impact of mutations and modifications on molecular interactions. Nucleic Acids Res. 43:e10. 10.1093/nar/gku1094 25392414 PMC4333368

[B5] Blume M. Nitzsche R. Sternberg U. Gerlic M. Masters S. L. Gupta N.. (2015). A *Toxoplasma gondii* gluconeogenic enzyme contributes to robust central carbon metabolism and is essential for replication and virulence. Cell Host Microbe 18, 210–220. 10.1016/j.chom.2015.07.008 26269956

[B6] Borchert N. Krug K. Gnad F. Sinha A. Sommer R. J. Macek B. (2012). Phosphoproteome of *Pristionchus pacificus* provides insights into architecture of signaling networks in nematode models. Mol. Cell. Proteomics 11, 1631–1639. 10.1074/mcp.M112.022103 22923814 PMC3518110

[B7] Cheng W. Liu F. Li M. Hu X. Chen H. Pappoe F.. (2015). Variation detection based on next-generation sequencing of type Chinese 1 strains of *Toxoplasma gondii* with different virulence from China. BMC Genomics 16:888. 10.1186/s12864-015-2106-z 26518334 PMC4628340

[B8] Choi S. H. Kim T. Y. Park S. G. Cha G. H. Shin D. W. Chai J. Y.. (2010). Proteomic analysis of *Toxoplasma gondii* KI-1 tachyzoites. Korean J. Parasitol. 48, 195–201. 10.3347/kjp.2010.48.3.195 20877497 PMC2945793

[B9] Delorme V. Cayla X. Faure G. Garcia A. Tardieux I. (2003). Actin dynamics is controlled by a casein kinase II and phosphatase 2C interplay on *Toxoplasma gondii* toxofilin. Mol. Biol. Cell. 14, 1900–1912. 10.1091/mbc.E02-08-0462 12802063 PMC165085

[B10] Deshmukh A. S. Mitra P. Kolagani A. Gurupwar R. (2018). Cdk-related kinase 9 regulates RNA polymerase II mediated transcription in *Toxoplasma gondii*. Biochim. Biophys. Acta Gene Regul. Mech. 1861, 572–585. 10.1016/j.bbagrm.2018.02.004 29466697

[B11] Deshmukh A. S. Mitra P. Maruthi M. (2016). Cdk7 mediates RPB1-driven mRNA synthesis in *Toxoplasma gondii*. Sci. Rep. 6:35288. 10.1038/srep35288 27759017 PMC5069487

[B12] Dixon S. E. Stilger K. L. Elias E. V. Naguleswaran A. Sullivan W. J. Jr. (2010). A decade of epigenetic research in *Toxoplasma gondii*. Mol. Biochem. Parasitol. 173, 1–9. 10.1016/j.molbiopara.2010.05.001 20470832 PMC2886187

[B13] Du J. An R. Chen L. Shen Y. Chen Y. Cheng L.. (2014). *Toxoplasma gondii* virulence factor ROP18 inhibits the host NF-κB pathway by promoting p65 degradation. J. Biol. Chem. 289, 12578–12592. 10.1074/jbc.M113.544718 24648522 PMC4007449

[B14] Elsheikha H. M. (2008). Congenital toxoplasmosis: priorities for further health promotion action. Public Health 122, 335–353. 10.1016/j.puhe.2007.08.009 17964621

[B15] Finn R. D. Coggill P. Eberhardt R. Y. Eddy S. R. Mistry J. Mitchell A. L.. (2016). The Pfam protein families database: towards a more sustainable future. Nucleic Acids Res. 44, D279–D285. 10.1093/nar/gkv1344 26673716 PMC4702930

[B16] Glibert P. Meert P. Van Steendam K. Van Nieuwerburgh F. De Coninck D. Martens L.. (2015). Phospho-iTRAQ: assessing isobaric labels for the large-scale study of phosphopeptide stoichiometry. J. Proteome Res. 14, 839–849. 10.1016/j.dib.2015.04.012 25510630

[B17] He W. Staples D. Smith C. Fisher C. (2003). Direct activation of cyclin-dependent kinase 2 by human papillomavirus E7. J. Virol. 77, 10566–10574. 10.1128/jvi.77.19.10566-10574.2003 12970441 PMC228519

[B18] Howe D. K. Sibley L. D. (1995). *Toxoplasma gondii* comprises three clonal lineages: correlation of parasite genotype with human disease. J. Infect. Dis. 172, 1561–1566. 10.1093/infdis/172.6.1561 7594717

[B19] Huttlin E. L. Jedrychowski M. P. Elias J. E. Goswami T. Rad R. Beausoleil S. A.. (2010). A tissue-specific atlas of mouse protein phosphorylation and expression. Cell 143, 1174–1189. 10.1016/j.cell.2010.12.001 21183079 PMC3035969

[B20] Ivanova D. L. Fatima R. Gigley J. P. (2016). Comparative analysis of conventional natural killer cell responses to acute infection with *Toxoplasma gondii* strains of different virulence. Front. Immunol. 7:347. 10.3389/fimmu.2016.00347 27721814 PMC5033988

[B21] Jan G. Delorme V. Saksouk N. Abrivard M. Gonzalez V. Cayla X.. (2009). A *Toxoplasma* type 2C serine-threonine phosphatase is involved in parasite growth in the mammalian host cell. Microbes Infect. 11, 935–945. 10.1016/j.micinf.2009.06.002 19563907

[B22] Jia Y. Marq J. B. Bisio H. Jacot D. Mueller C. Yu L.. (2017). Crosstalk between PKA and PKG controls pH-dependent host cell egress of *Toxoplasma gondii*. EMBO J. 36, 3250–3267. 10.15252/embj.201796794 29030485 PMC5666616

[B23] Jiang H. H. Huang S. Y. Zhou D. H. Zhang X. X. Su C. Deng S. Z.. (2013). Genetic characterization of *Toxoplasma gondii* from pigs from different localities in China by PCR-RFPL. Parasit. Vectors 6:227. 10.1186/1756-3305-6-227 23919620 PMC3750303

[B24] Joyce B. R. Konrad C. Wek R. C. Sullivan W. J. Jr. (2011). Translation control is critical during acute and chronic stages of toxoplasmosis infection. Expert Rev. Anti Infect. Ther. 9, 1–3. 10.1586/eri.10.146 21171869

[B25] Khan A. Dubey J. P. Su C. Ajioka J. W. Rosenthal B. M. Sibley L. D. (2011). Genetic analyses of atypical *Toxoplasma gondii* strains reveal a fourth clonal lineage in North America. Int. J. Parasitol. 41, 645–655. 10.1016/j.ijpara.2011.01.005 21320505 PMC3081397

[B26] Khan A. Taylor S. Ajioka J. W. Rosenthal B. M. Sibley L. D. (2009). Selection at a single locus leads to widespread expansion of *Toxoplasma gondii* lineages that are virulent in mice. PLoS Genet. 5:e1000404. 10.1371/journal.pgen.1000404 19266027 PMC2644818

[B27] Khan F. Tang J. Qin C. L. Kim K. (2002). Cyclin-dependent kinase TPK2 is a critical cell cycle regulator in *Toxoplasma gondii*. Mol. Microbiol. 45, 321–332. 10.1046/j.1365-2958.2002.03026.x 12123447

[B28] Li M. Mo X. W. Wang L. Chen H. Luo Q. L. Wen H. Q.. (2014). Phylogeny and virulence divergency analyses of *Toxoplasma gondii* isolates from China. Parasit. Vectors 7:133. 10.1186/1756-3305-7-133 24678633 PMC3986613

[B29] Lim D. C. Cooke B. M. Doerig C. Saeij J. P. (2012). *Toxoplasma* and *Plasmodium* protein kinases: roles in invasion and host cell remodelling. Int. J. Parasitol. 42, 21–32. 10.1016/j.ijpara.2011.11.007 22154850 PMC3428259

[B30] Lim W. A. Pawson T. (2010). Phosphotyrosine signaling: evolving a new cellular communication system. Cell 142, 661–667. 10.1016/j.cell.2010.08.023 20813250 PMC2950826

[B31] Lorenzi H. Khan A. Behnke M. S. Namasivayam S. Swapna L. S. Hadjithomas M.. (2016). Local admixture of amplified and diversified secreted pathogenesis determinants shapes mosaic *Toxoplasma gondii* genomes. Nat. Commun. 7:10147. 10.1038/ncomms10147 26738725 PMC4729833

[B32] Lun Z. R. Lai D. H. Wen Y. Z. Zheng L. L. Shen J. L. Yang T. B.. (2015). Cancer in the parasitic protozoans *Trypanosoma brucei* and *Toxoplasma gondii*. Proc. Natl. Acad. Sci. U.S.A. 112, 8835–8842. 10.1073/pnas.1502599112 26195778 PMC4517281

[B33] Mithoe S. C. Menke F. L. (2011). Phosphoproteomics perspective on plant signal transduction and tyrosine phosphorylation. Phytochemistry 72, 997–1006. 10.1016/j.phytochem.2010.12.009 21315387

[B34] Molestina R. E. Sinai A. P. (2005). Detection of a novel parasite kinase activity at the *Toxoplasma gondii* parasitophorous vacuole membrane capable of phosphorylating host IkappaBalpha. Cell. Microbiol. 7, 351–362. 10.1111/j.1462-5822.2004.00463.x 15679838

[B35] Nakayasu E. S. Tempel R. Cambronne X. A. Petyuk V. A. Jones M. B. Gritsenko M. A.. (2013). Comparative phosphoproteomics reveals components of host cell invasion and post-transcriptional regulation during *Francisella* infection. Mol. Cell. Proteomics 12, 3297–3309. 10.1074/mcp.M113.029850 23970565 PMC3820940

[B36] Niedelman W. Gold D. A. Rosowski E. E. Sprokholt J. K. Lim D. Farid Arenas A. (2012). The rhoptry proteins ROP18 and ROP5 mediate *Toxoplasma gondii* evasion of the murine, but not the human, interferon-gamma response. PLoS Pathog. 8:e1002784 10.1371/journal.ppat.1002784 22761577 PMC3386190

[B37] Olsen J. V. Blagoev B. Gnad F. Macek B. Kumar C. Mortensen P.. (2006). Global, *in vivo*, and site-specific phosphorylation dynamics in signaling networks. Cell 127, 635–648. 10.1016/j.cell.2006.09.026 17081983

[B38] Ong Y. C. Reese M. L. Boothroyd J. C. (2010). *Toxoplasma* rhoptry protein 16 (ROP16) subverts host function by direct tyrosine phosphorylation of STAT6. J. Biol. Chem. 285, 28731–28740. 10.1074/jbc.M110.112359 20624917 PMC2937901

[B39] Pawson T. Scott J. D. (2005). Protein phosphorylation in signaling −50 years and counting. Trends Biochem. Sci. 30, 286–290. 10.1016/j.tibs.2005.04.013 15950870

[B40] Pearlman S. M. Serber Z. Ferrell J. E. Jr. (2011). A mechanism for the evolution of phosphorylation sites. Cell 147, 934–946. 10.1016/j.cell.2011.08.052 22078888 PMC3220604

[B41] Peixoto L. Chen F. Harb O. S. Davis P. H. Beiting D. P. Brownback C. S.. (2010). Integrative genomic approaches highlight a family of parasite-specific kinases that regulate host responses. Cell Host Microbe 8, 208–218. 10.1016/j.chom.2010.07.004 20709297 PMC2963626

[B42] Pi E. Qu L. Hu J. Huang Y. Qiu L. Lu H.. (2016). Mechanisms of soybean roots' tolerances to salinity revealed by proteomic and phosphoproteomic comparisons between two cultivars. Mol. Cell. Proteomics 15, 266–288. 10.1074/mcp.M115.051961 26407991 PMC4762511

[B43] Pittman K. J. Aliota M. T. Knoll L. J. (2014). Dual transcriptional profiling of mice and *Toxoplasma gondii* during acute and chronic infection. BMC Genomics 15:806. 10.1186/1471-2164-15-806 25240600 PMC4177681

[B44] Prandovszky E. Gaskell E. Martin H. Dubey J. P. Webster J. P. McConkey G. A. (2011). The neurotropic parasite *Toxoplasma gondii* increases dopamine metabolism. PLoS ONE 6:e23866. 10.1371/journal.pone.0023866 21957440 PMC3177840

[B45] Ramírez-Flores C. J. Cruz-Mirón R. Arroyo R. Mondragón-Castelán M. E. Nopal-Guerrero T. González-Pozos S.. (2019). Characterization of metalloproteases and serine proteases of *Toxoplasma gondii* tachyzoites and their effect on epithelial cells. Parasitol. Res. 118, 289–306. 10.1007/s00436-018-6163-5 30506516

[B46] Reese M. L. Zeiner G. M. Saeij J. P. Boothroyd J. C. Boyle J. P. (2011). Polymorphic family of injected pseudokinases is paramount in *Toxoplasma* virulence. Proc. Natl. Acad. Sci. U.S.A. 108, 9625–9630. 10.1073/pnas.1015980108 21436047 PMC3111280

[B47] Saeij J. P. Boyle J. P. Boothroyd J. C. (2005). Differences among the three major strains of *Toxoplasma gondii* and their specific interactions with the infected host. Trends Parasitol. 21, 476–481. 10.1016/j.pt.2005.08.001 16098810

[B48] Saeij J. P. Boyle J. P. Coller S. Taylor S. Sibley L. D. Brooke-Powell E. T. (2006). Polymorphic secreted kinases are key virulence factors in toxoplasmosis. Science. 2314, 1780–1783. 10.1126/science.1133690 PMC2646183 17170306

[B49] Saeij J. P. Coller S. Boyle J. P. Jerome M. E. White M. W.. (2007). *Toxoplasma* co-opts host gene expression by injection of a polymorphic kinase homologue. Nature 445, 324–327. 10.1038/nature05395 17183270 PMC2637441

[B50] Saha A. Robertson E. S. (2011). Epstein-Barr virus-associated B-cell lymphomas: pathogenesis and clinical outcomes. Clin. Cancer Res. 17, 3056–3063. 10.1158/1078-0432 21372216 PMC4287361

[B51] Schwartz D. Gygi S. P. (2005). An iterative statistical approach to the identification of protein phosphorylation motifs from large-scale datasets. Nat. Biotechnol. 23, 1391–1398. 10.1038/nbt1146 16273072

[B52] Shaw M. K. Roos D. S. Tilney L. G. (2002). Cysteine and serine protease inhibitors block intracellular development and disrupt the secretory pathway of *Toxoplasma gondii*. Microbes Infect. 4, 119–132. 10.1016/S1286-4579(01)01520-9 11880042

[B53] Shen B. Sibley L. D. (2014). *Toxoplasma* aldolase is required for metabolism but dispensable for host-cell invasion. Proc. Natl. Acad. Sci. U.S.A. 111, 3567–3572. 10.1073/pnas.1315156111 24550496 PMC3948255

[B54] Shwab E. K. Zhu X. Q. Majumdar D. Pena H. F. Gennari S. M. Dubey J. P.. (2014). Geographical patterns of *Toxoplasma gondii* genetic diversity revealed by multilocus PCR-RFLP genotyping. Parasitology 141, 453–461. 10.1017/S0031182013001844 24477076

[B55] Sibley L. D. Ajioka J. W. (2008). Population structure of *Toxoplasma gondii*: clonal expansion driven by infrequent recombination and selective sweeps. Annu. Rev. Microbiol. 62, 329–351. 10.1146/annurev.micro.62.081307.162925 18544039

[B56] Sibley L. D. Boothroyd J. C. (1992). Virulent strains of *Toxoplasma gondii* comprise a single clonal lineage. Nature 359, 82–85. 10.1038/359082a0 1355855

[B57] Sibley L. D. Khan A. Ajioka J. W. Rosenthal B. M. (2009a). Genetic diversity of *Toxoplasma gondii* in animals and humans. Philos. Trans. R. Soc. Lond. B. Biol. Sci. 364, 2749–2761. 10.1098/rstb.2009.0087 19687043 PMC2865090

[B58] Sibley L. D. Qiu W. Fentress S. Taylor S. J. Khan A. Hui R. (2009b). Forward genetics in *Toxoplasma gondii* reveals a family of rhoptry kinases that mediates pathogenesis. Eukaryotic Cell 8, 1085–1093. 10.1128/EC.00107-09 19465561 PMC2725553

[B59] Sievers F. Wilm A. Dineen D. Gibson T. J. Karplus K. Li W.. (2011). Fast, scalable generation of high-quality protein multiple sequence alignments using clustal omega. Mol. Syst. Biol. 7:539. 10.1038/msb.2011.75 21988835 PMC3261699

[B60] Solari F. A. Kollipara L. Sickmann A. Zahedi R. P. (2016). Two birds with one stone: parallel quantification of proteome and phosphoproteome using iTRAQ. Methods Mol. Biol. 1394, 25–41. 10.1007/978-1-4939-3341-9_3 26700039

[B61] Starnes G. L. Coincon M. Sygusch J. Sibley L. D. (2009). Aldolase is essential for energy production and bridging adhesin-actin cytoskeletal interactions during parasite invasion of host cells. Cell Host Microbe 5, 353–364. 10.1016/j.chom.2009.03.005 19380114 PMC2683947

[B62] Steinfeldt T. Könen-Waisman S. Tong L. Pawlowski N. Lamkemeyer T. Sibley L. D.. (2015). Correction: Phosphorylation of mouse immunity-related GTPase (IRG) resistance proteins is an evasion strategy for virulent *Toxoplasma gondii*. PLoS Biol. 13:e1002199. 10.1371/journal.pbio.1000576 26158675 PMC4497683

[B63] Sugi T. Tu V. Ma Y. Tomita T. Weiss L. M. (2017). *Toxoplasma gondii* requires glycogen phosphorylase for balancing amylopectin storage and for efficient production of brain cysts. MBio 8:e01289-17. 10.1128/mBio.01289-17 28851850 PMC5574715

[B64] Sullivan W. J. Jr. Jeffers V. (2012). Mechanisms of *Toxoplasma gondii* persistence and latency. FEMS Microbiol. Rev. 36, 717–733. 10.1111/j.1574-6976.2011.00305.x 22091606 PMC3319474

[B65] Taylor S. Barragan A. Su C. Fux B. Fentress S. J. Tang K.. (2006). A secreted serine-threonine kinase determines virulence in the eukaryotic pathogen *Toxoplasma gondii*. Science 314, 1776–1780. 10.1126/science.1133643 17170305

[B66] Thirugnanam S. Rout N. Gnanasekar M. (2013). Possible role of *Toxoplasma gondii* in brain cancer through modulation of host microRNAs. Infect. Agents Cancer. 8:8. 10.1186/1750-9378-8-8 23391314 PMC3583726

[B67] Treeck M. Sanders J. L. Elias J. E. Boothroyd J. C. (2011). The phosphoproteomes of *Plasmodium falciparum* and *Toxoplasma gondii* reveal unusual adaptations within and beyond the parasites' boundaries. Cell Host Microbe 10, 410–419. 10.1016/j.chom.2011.09.004 22018241 PMC3254672

[B68] Treeck M. Sanders J. L. Gaji R. Y. LaFavers K. A. Child M. A. Arrizabalaga G.. (2014). The calcium-dependent protein kinase 3 of *Toxoplasma* influences basal calcium levels and functions beyond egress as revealed by quantitative phosphoproteome analysis. PLoS Pathog. 10:e1004197. 10.1371/journal.ppat.1004197 24945436 PMC4063958

[B69] Vescovo T. Refolo G. Vitagliano G. Fimia G. M. Piacentini M. (2016). Molecular mechanisms of hepatitis C virus-induced hepatocellular carcinoma. Clin. Microbiol. Infect. 22, 853–861. 10.1016/j.cmi.2016.07.019 27476823

[B70] Wang L. Chen H. Liu D. Huo X. Gao J. Song X.. (2013). Genotypes and mouse virulence of *Toxoplasma gondii* isolates from animals and humans in China. PLoS ONE 8:e53483. 10.1371/journal.pone.0053483 23308233 PMC3538538

[B71] Wang L. He L. Y. Meng D. D. Chen Z. W. Wen H. Fang G. S.. (2015). Seroprevalence and genetic characterization of *Toxoplasma gondii* in cancer patients in Anhui Province, Eastern China. Parasit. Vectors 8:162. 10.1186/s13071-015-0778-5 25889184 PMC4379604

[B72] Wei F. Wang W. Liu Q. (2013). Protein kinases of *Toxoplasma gondii*: functions and drug targets. Parasitol. Res. 112, 2121–2129. 10.1007/s00436-013-3451-y 23681193

[B73] Yang N. Farrell A. Niedelman W. Melo M. Lu D. Julien L.. (2013). Genetic basis for phenotypic differences between different *Toxoplasma gondii* type I strains. BMC Genomics 14:467. 10.1186/1471-2164-14-467 23837824 PMC3710486

[B74] Yeoh L. M. Goodman C. D. Hall N. E. van Dooren G. G. McFadden G. I. Ralph S. A. (2015). A serine-arginine-rich (SR) splicing factor modulates alternative splicing of over a thousand genes in *Toxoplasma gondii*. Nucleic Acids Res. 43, 4661–4675. 10.1093/nar/gkv311 25870410 PMC4482073

[B75] Zhou D. H. Wang Z. X. Zhou C. X. He S. Elsheikha H. M. Zhu X. Q. (2017). Comparative proteomic analysis of virulent and avirulent strains of *Toxoplasma gondii* reveals strain-specific patterns. Oncotarget 8, 80481–80491. 10.18632/oncotarget.19077 29113319 PMC5655214

[B76] Zhou D. H. Zhao F. R. Nisbet A. J. Xu M. J. Song H. Q. Lin R. Q.. (2014). Comparative proteomic analysis of different *Toxoplasma gondii* genotypes by two-dimensional fluorescence difference gel electrophoresis combined with mass spectrometry. Electrophoresis 35, 533–545. 10.1002/elps.201300044 24166805

