# Supplementary figures and images for: iTRAQ-Based Global Phosphoproteomics Reveals Novel Molecular Differences Between Toxoplasma gondii Strains of Different Genotypes
Source: Front Cell Infect Microbiol. 2019 Aug 23;9:307. doi: 10.3389/fcimb.2019.00307 (PMC6716450; doi:10.3389/fcimb.2019.00307)

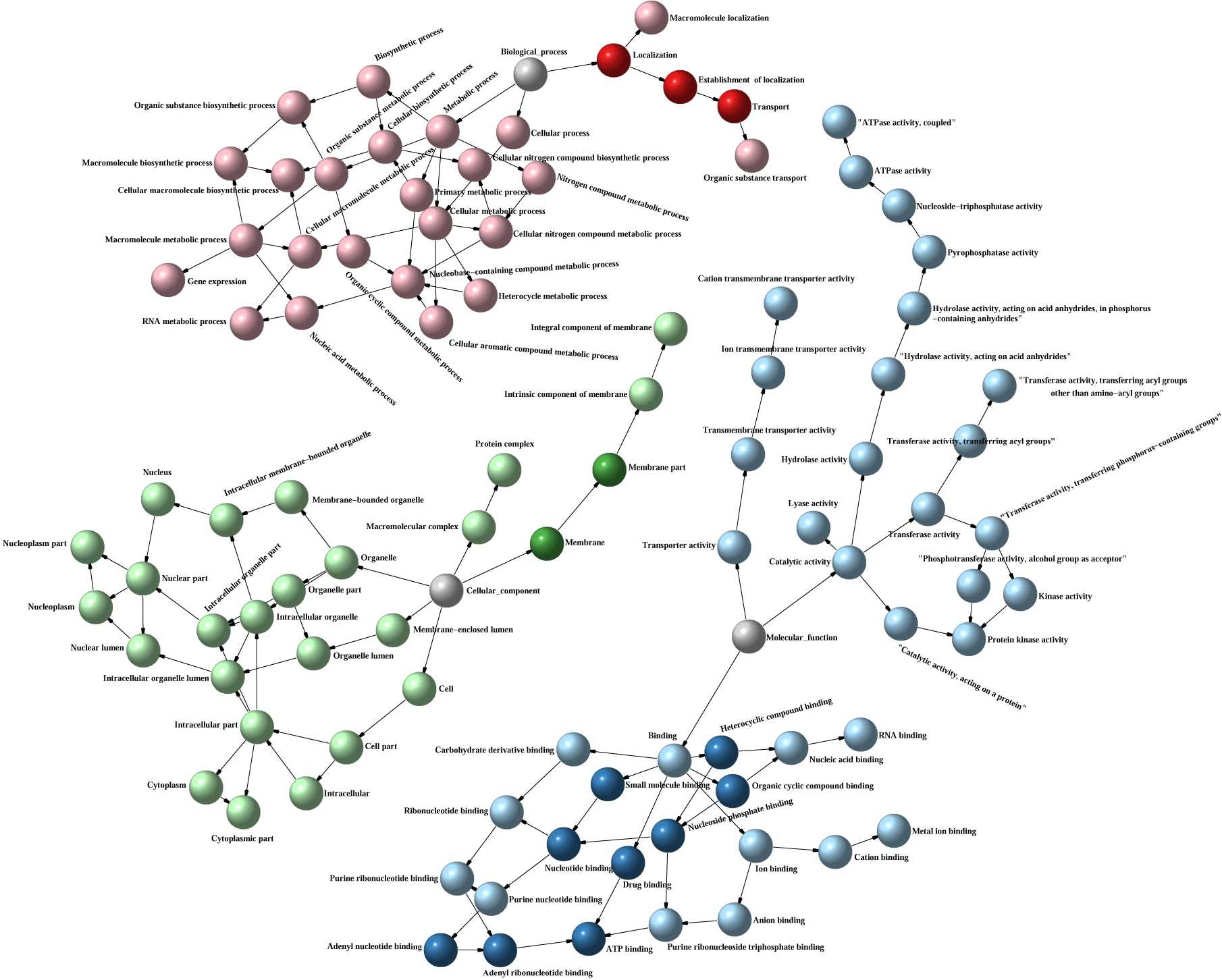

Supplement: Figure S1 — GO function network of upregulated phosphoproteins in RH strain when comparing RH/PRU strains. The clusters coincide with the GO functional categories of the DEPs and are color-coded as indicated. Red, green and blue clusters denote GO terms related to biological process, cellular component and molecular function, respectively. The dark colored nodes represent significantly enriched GO terms (p-value ≤ 0.05). The solid and dashed arrows between nodes represent direct and indirect associations between GO terms, respectively. [file Image_1.TIF]

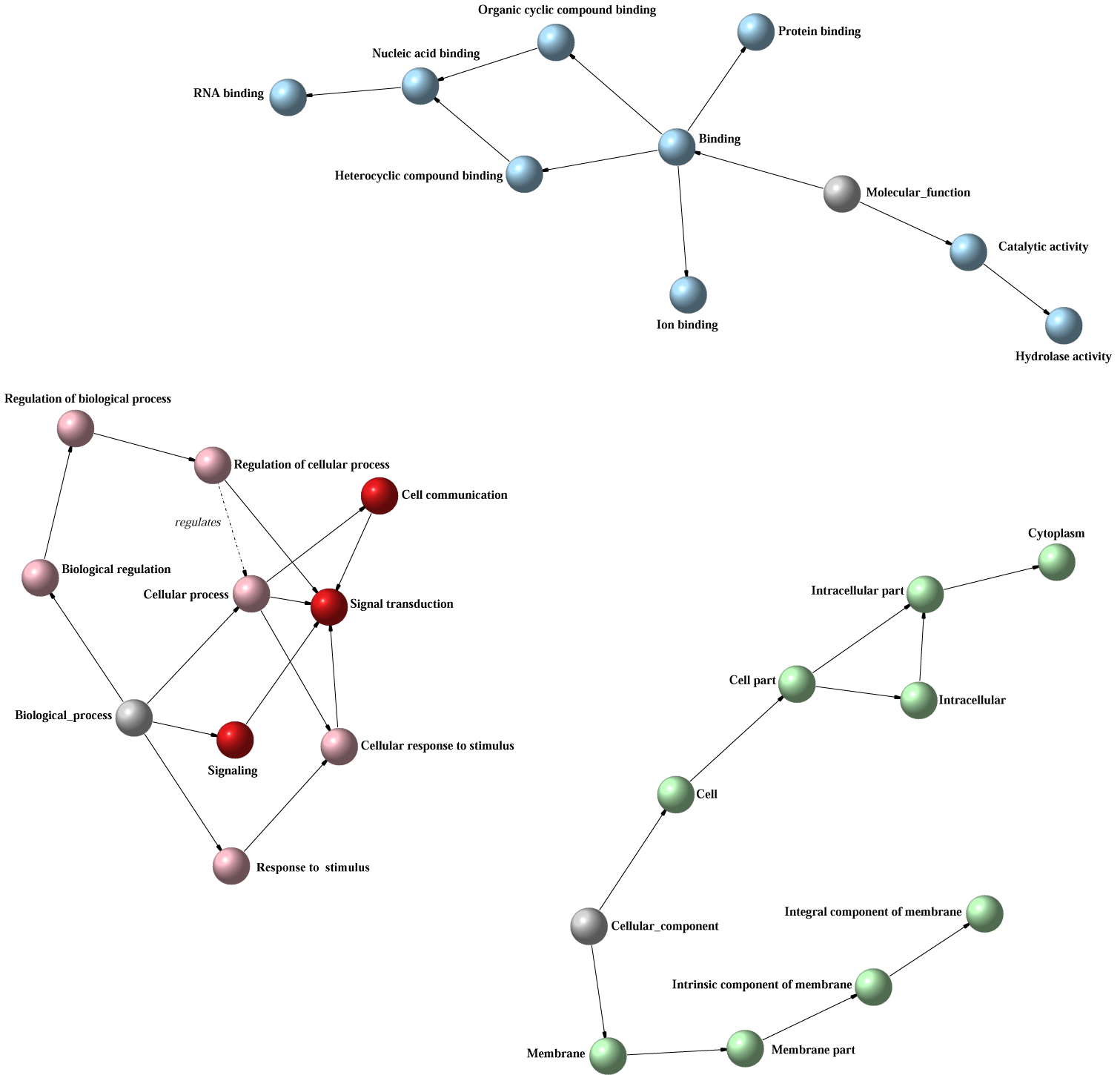

Supplement: Figure S2 — GO function network of downregulated phosphoproteins in RH strain when comparing RH/PRU strains. The clusters coincide with the GO functional categories of the DEPs and are color-coded as indicated. Red, green and blue clusters denote GO terms related to biological process, cellular component and molecular function, respectively. The dark colored nodes represent significantly enriched GO terms (p-value ≤ 0.05). The solid and dashed arrows between nodes represent direct and indirect associations between GO terms, respectively. [file Image_2.TIF]

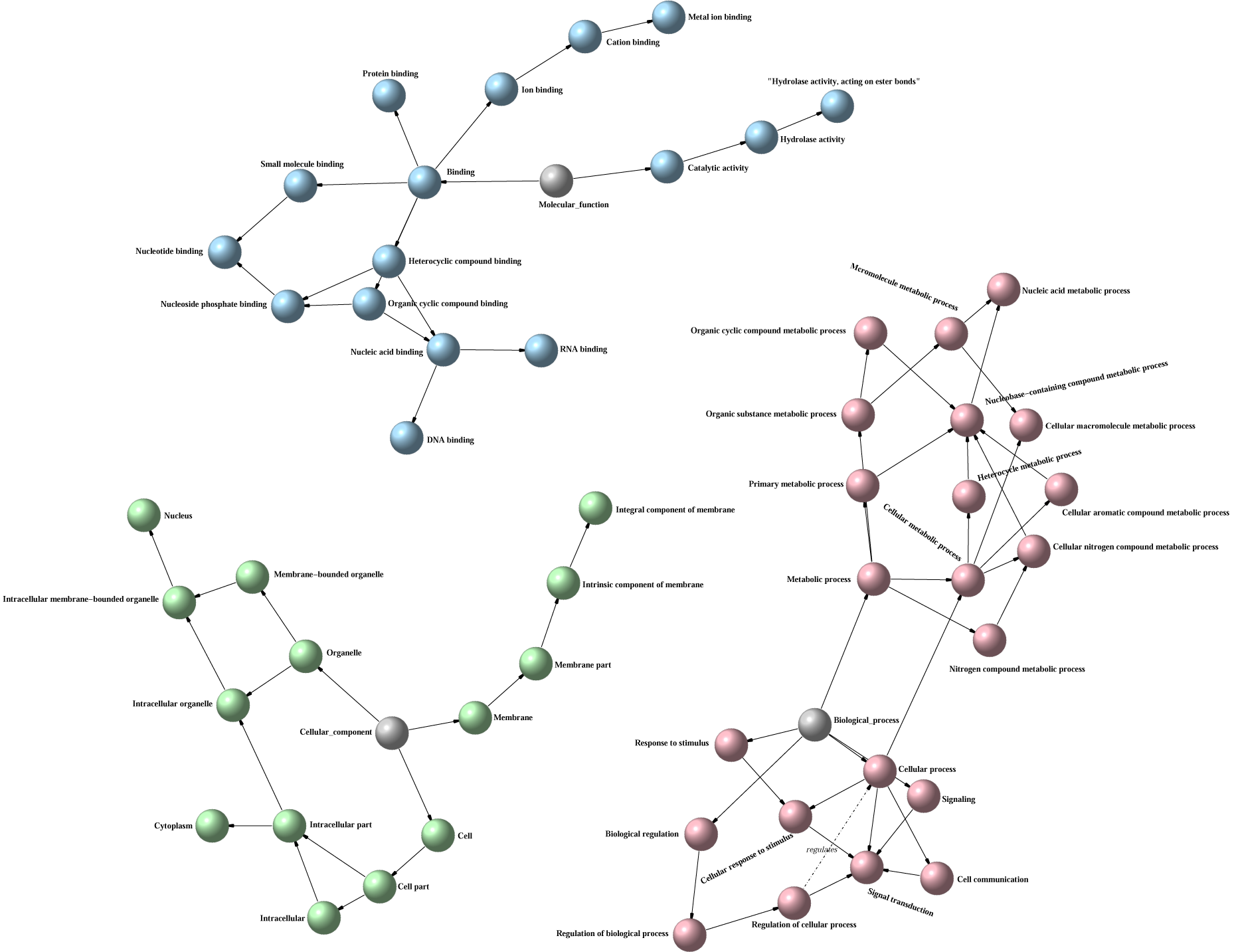

Supplement: Figure S3 — GO function network of upregulated phosphoproteins in PRU strain when comparing PRU/PYS strains. The clusters coincide with the GO functional categories of the DEPs and are color-coded as indicated. Red, green, and blue clusters denote GO terms related to biological process, cellular component and molecular function, respectively. The solid and dashed arrows between nodes represent direct and indirect associations between GO terms, respectively. [file Image_3.TIF]

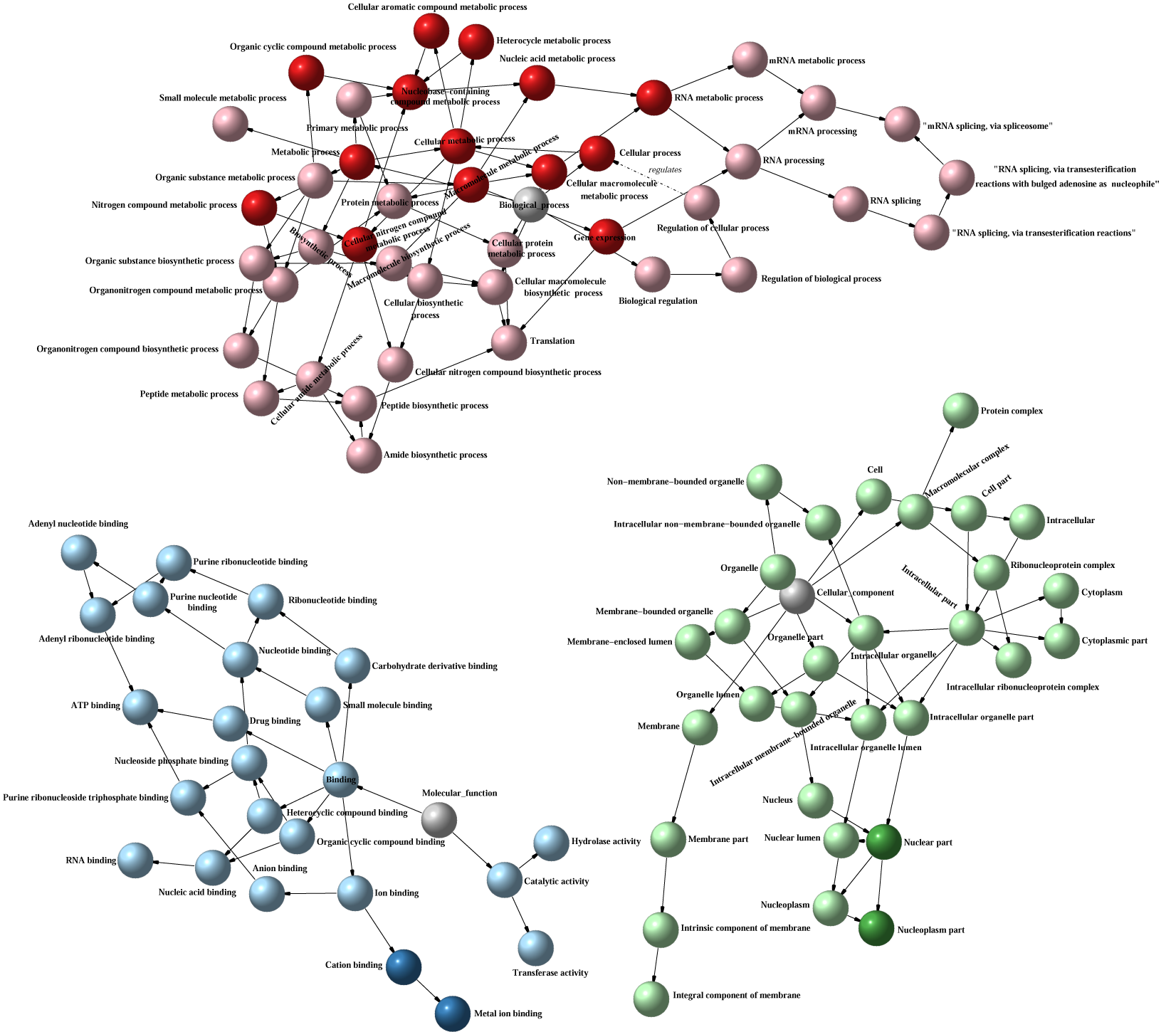

Supplement: Figure S4 — GO function network of downregulated phosphoproteins in PRU strain when comparing PRU/PYS strains. The clusters coincide with the GO functional categories of the DEPs and are color-coded as indicated. Red, green, and blue clusters denote GO terms related to biological process, cellular component and molecular function, respectively. The dark colored nodes represent significantly enriched GO terms (p-value ≤ 0.05). The solid and dashed arrows between nodes represent direct and indirect associations between GO terms, respectively. [file Image_4.TIF]

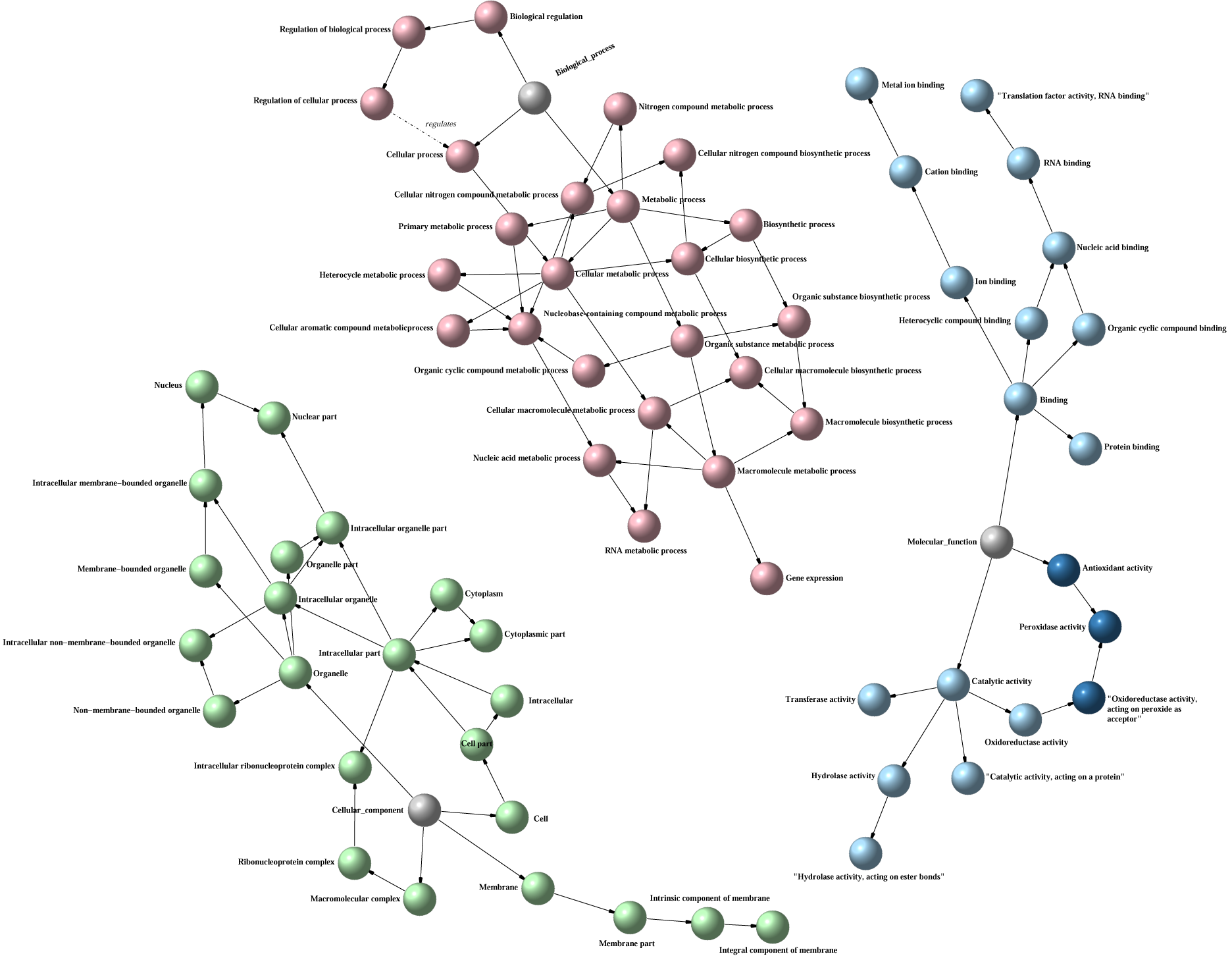

Supplement: Figure S5 — GO-network of upregulated phosphoproteins in PYS strain when comparing PYS/RH strains. Red nodes, green nodes, and blue nodes denote GO terms under biological process, GO terms under cellular component and GO terms under molecular function, respectively. Nodes with deeper red, deeper green, and deeper blue represent significantly enriched GO terms (p-value ≤ 0.05) under biological process, cellular component and molecular function, respectively. Edges (solid and dotted arrows between nodes) represent direct (solid lines) and indirect (dashed lines) interactions between molecules. [file Image_5.TIF]

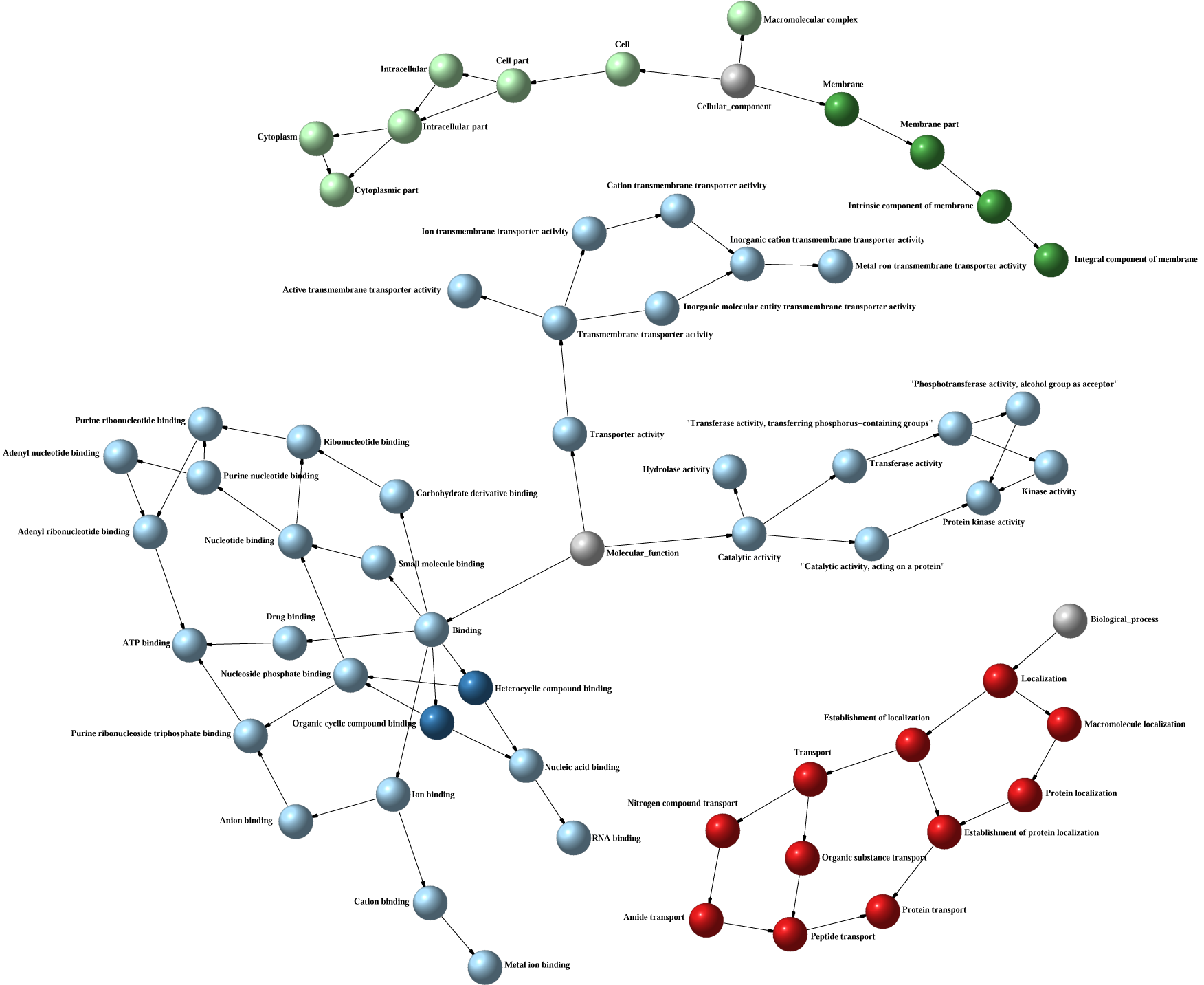

Supplement: Figure S6 — GO function network of downregulated phosphoproteins in PYS strain when comparing PYS/RH strains. The clusters coincide with the GO functional categories of the DEPs and are color-coded as indicated. Red, green, and blue clusters denote GO terms related to biological process, cellular component and molecular function, respectively. The dark colored nodes represent significantly enriched GO terms (p-value ≤ 0.05). The solid and dashed arrows between nodes represent direct and indirect associations between GO terms, respectively. [file Image_6.TIF]

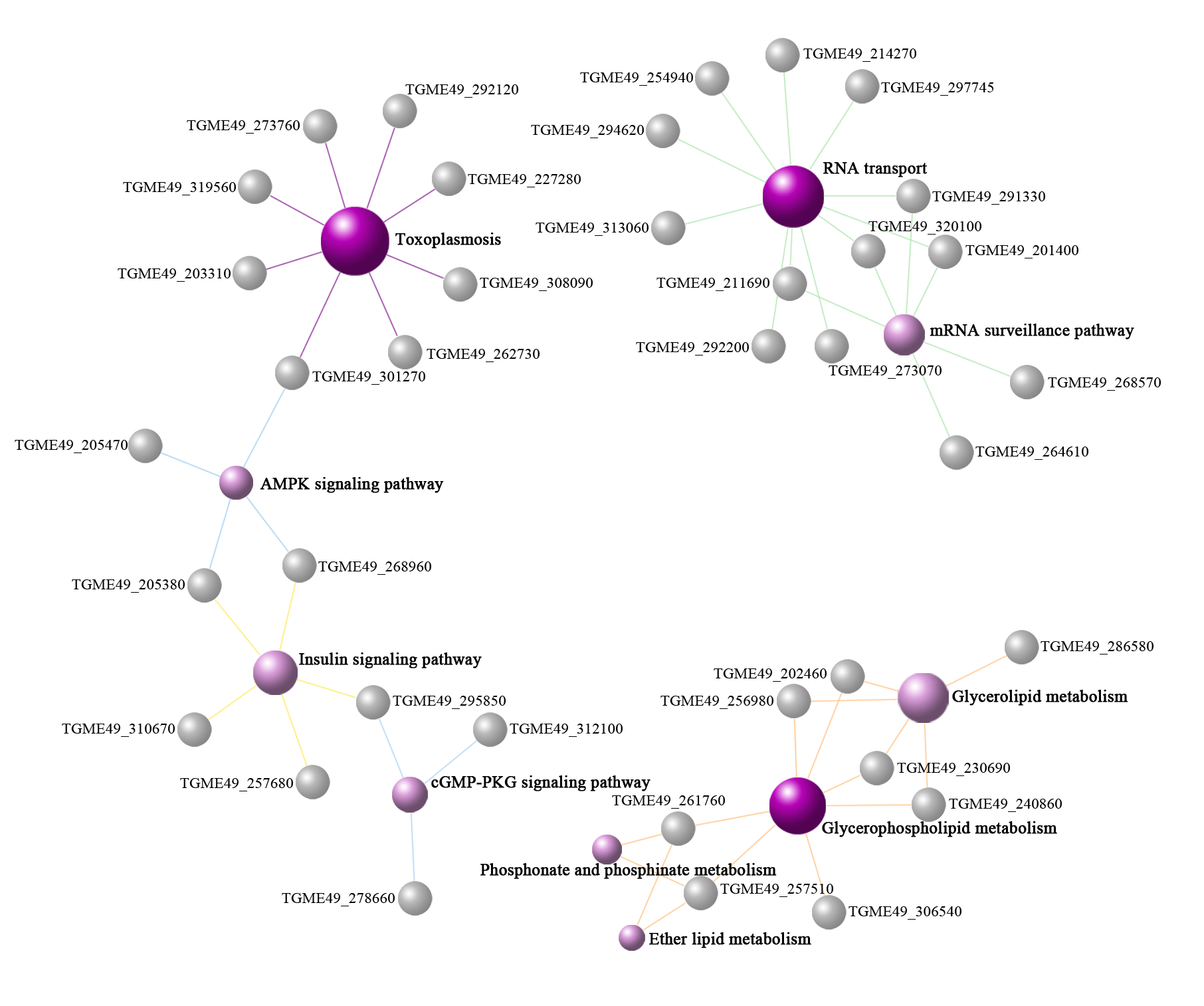

Supplement: Figure S7 — KEGG network of differentially expressed phosphoproteins in RH strain when comparing RH/PRU strains. Light purple nodes represent non-significantly enriched pathways and deep purple nodes indicate significantly enriched pathways (p-value ≤ 0.05). The gray nodes surrounding purple nodes denote differentially expressed phosphoproteins. [file Image_7.TIF]

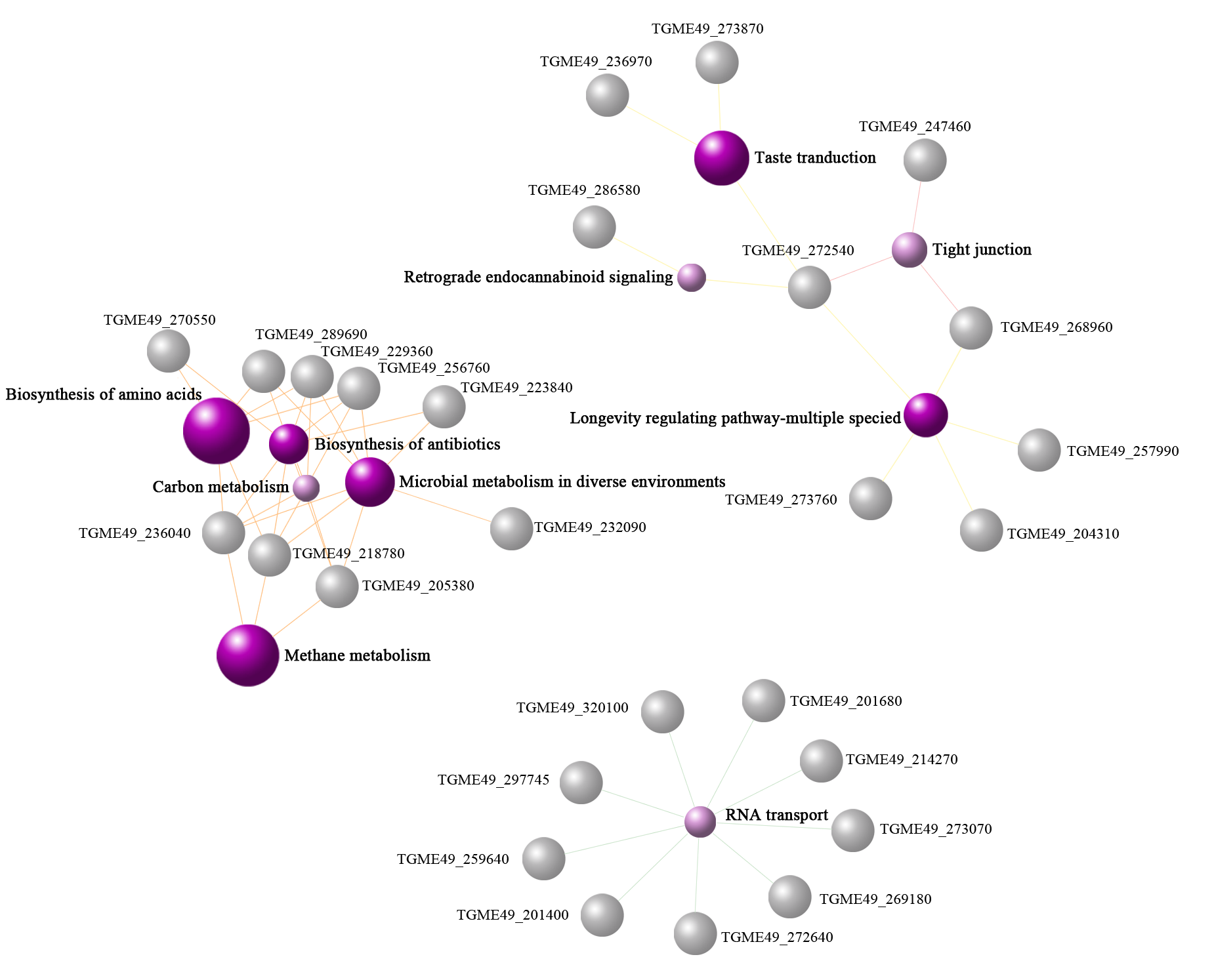

Supplement: Figure S8 — KEGG network of differentially expressed phosphoproteins in PRU strain when comparing PRU/PYS strains. Light purple nodes represent non-significantly enriched pathways and deep purple nodes indicate significantly enriched pathways (p-value ≤ 0.05). The gray nodes surrounding purple nodes denote differentially expressed phosphoproteins. [file Image_8.TIF]

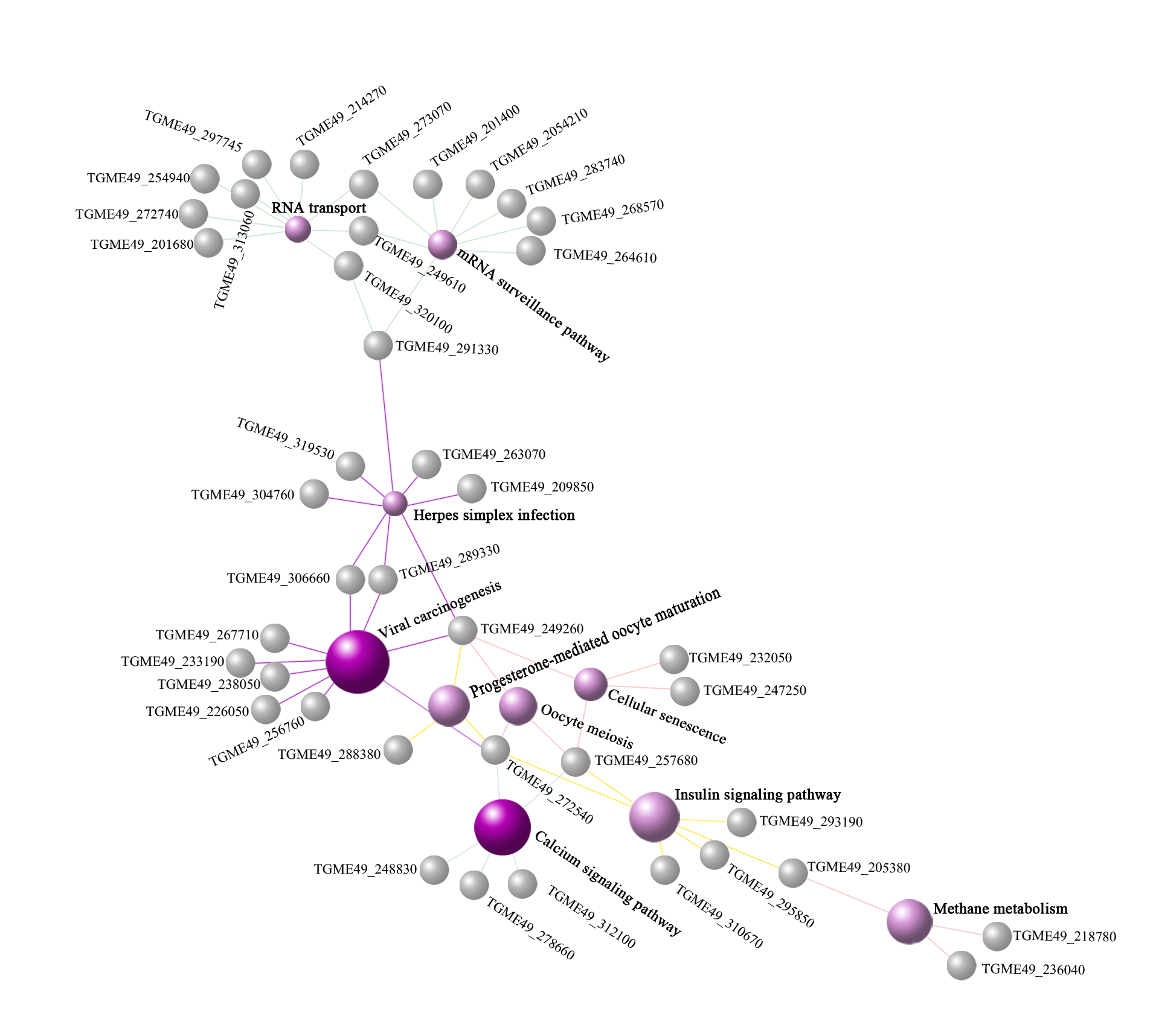

Supplement: Figure S9 — KEGG network of differentially expressed phosphoproteins in PYS strain when comparing PYS/RH strains. Light purple nodes represent non-significantly enriched pathways and deeper purple nodes indicated significantly enriched pathways (p-value ≤ 0.05). The gray nodes surrounding purple nodes denote differentially expressed phosphoproteins. [file Image_9.TIF]

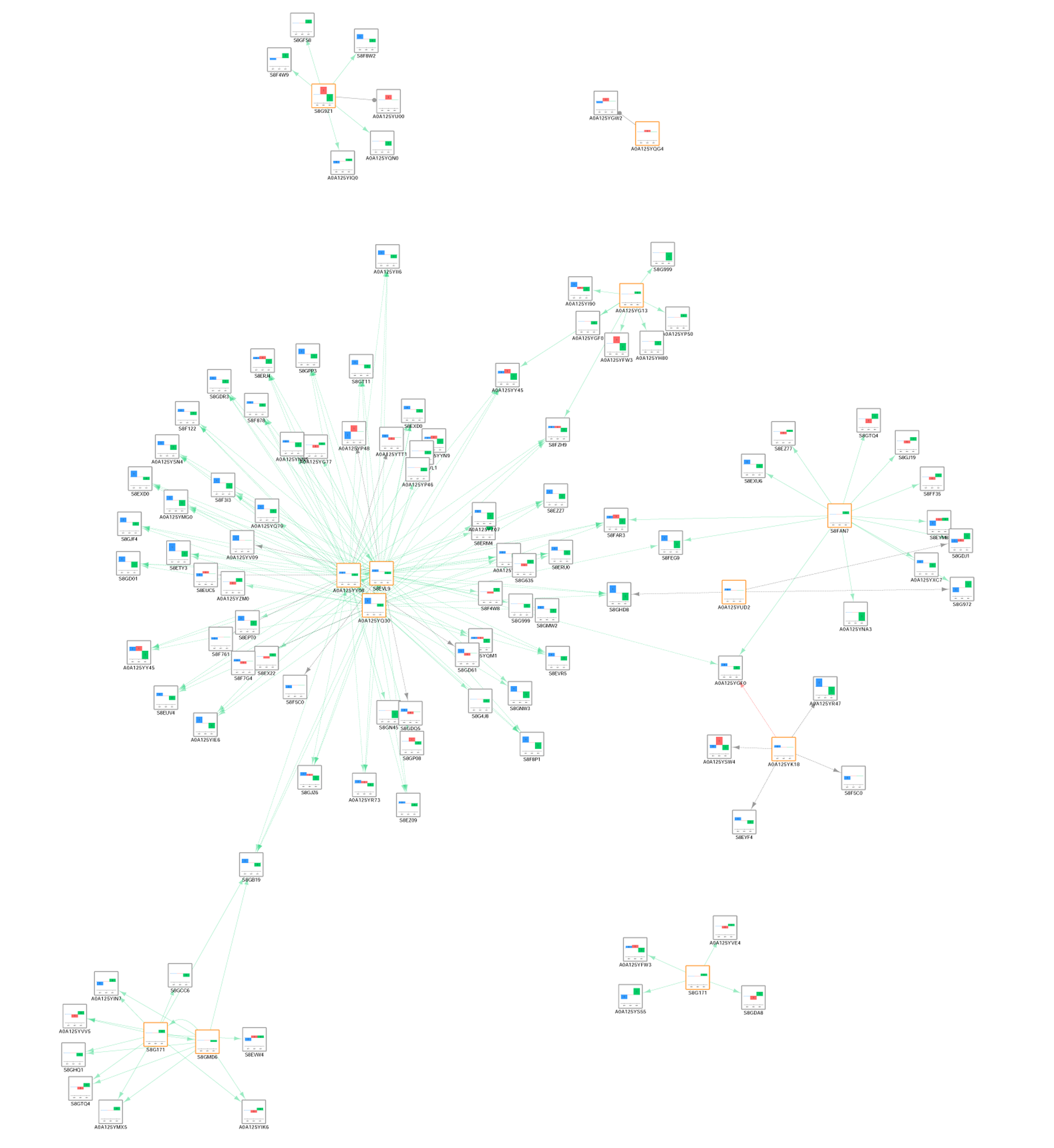

Supplement: Figure S10 — Network of kinase-phosphopeptide associations and potential phosphor-dependent interactions identified by correlation analysis. Log2 (fold changes) of phosphoproteins up/down-regulated in RH when comparing RH/PRU, PRU when comparing PRU/PYS and PYS when comparing PYS/RH were mapped in the nodes in blue, red, and green, respectively. The gray arrow indicates protein interactions that are predicted to be disabled by the phosphorylation and green arrows indicate protein interactions that are predicted to be enabled by the phosphorylation. [file Image_10.TIF]
